# Physiological and transcriptomic analyses of exogenous calcium in boosting nitrogen use efficiency via oxidative and resistance pathways in peanuts

**DOI:** 10.3389/fpls.2025.1629610

**Published:** 2026-01-27

**Authors:** Fengdan Xu, Liang Li, Xianzong Si, Yanyan Suo, Xiaolin Wang, Zhehui Zhang, Qian Li, Xiang Zhang

**Affiliations:** 1 Research Institute of Plant Nutrition and Resources & Environments/Henan Key Laboratory of Agricultural Resources and Environment, Henan Academy of Agricultural Sciences, Zhengzhou, Henan, China; 2 Henan Soil and Fertilizer Station, Department of Agriculture of Henan Province, Zhengzhou, Henan, China

**Keywords:** peanut, calcium, transcriptomic, oxidative, nitrogen efficiency

## Abstract

**Introduction:**

Peanuts (*Arachis hypogaea* L.) exhibit a high demand for calcium, second only to nitrogen and potassium, with calcium playing a critical role in their growth, development, and nitrogen fixation. However, the mechanisms underlying calcium-mediated regulation of peanut growth and nitrogen fixation remain poorly understood.

**Methods:**

In this study, we employed nitrogen-efficient (Puhua 66, Huayu 20) and nitrogen-inefficient (Puhua 28, Shanhua 14) peanut varieties in a two-year field experiment using a split-plot design. The main plots comprised two treatments: standard fertilization (CK) and calcium supplementation (Ca), while the sub-plots consisted of different peanut varieties. We analyzed growth parameters, physiological responses, and transcriptomic profiles.

**Results:**

Our results demonstrated that calcium application significantly increased malondialdehyde (MDA) content in leaves while reducing peroxidase (POD) activity, enhancing pod dry matter accumulation, and promoting earlier plant maturation. Additionally, calcium application elevated the activities of nitrate reductase (NR) and glutamine synthetase (GS) (P < 0.01), thereby improving nitrogen and calcium accumulation in pods, their allocation efficiency, and the overall utilization rates of nitrogen and calcium fertilizers. Transcriptomic analysis revealed 166 differentially expressed genes (DEGs) in nitrogen-efficient varieties and 343 DEGs in nitrogen-inefficient varieties under calcium supplementation, with 67 DEGs shared between the two groups. Functional annotation and qRT-PCR validation were performed on these DEGs.Furthermore, weighted gene co-expression network analysis (WGCNA) indicated that calcium supplementation significantly up-regulated genes associated with sucrose synthase, β-amylase, GTPase-activating proteins, light-harvesting chlorophyll-protein complexes (*Lhca2, Lhca3*), photosynthetic electron transport (*PetF, PetJ*), phosphatidylinositol phospholipase C2, inositol-3-phosphate synthase, TMV resistance protein, ABC transporters, ethylene-responsive transcription factors (*EIN1, EIN2, EIN3*), alkylamine oxidase, glutamate dehydrogenase, and aspartate synthase.

**Conclusion:**

These findings suggest that calcium application modulates carbohydrate metabolism, nitrogen assimilation, plant-pathogen interactions, and photosynthetic processes through differential gene expression, ultimately enhancing leaf physiological activity, dry matter partitioning, pod yield, and early maturation in peanuts.

## 1 Introduction

In recent years, the large-scale and intensive development of peanut cultivation has made continuous cropping increasingly common. However, long-term continuous cropping disrupts the equilibrium between soil nutrients, microbial communities, and crop requirements (Zhao et al., 2020). As a calcicolous plant, peanut requires up to 2~2.5 kg of calcium per 1000 kg of pod production (Yang et al., 2020). Yet, calcium fertilizer replenishment is frequently overlooked in agricultural practices, exacerbating soil calcium deficits (Yu et al., 2020). Calcium deficiency weakens peanut roots, causes pod shriveling, and increases the proportion of single-kernel pods, ultimately leading to empty pods and significantly reduced yields (Wu et al., 2020). Furthermore, calcium deficiency disrupts peanut metabolic processes, including reduced chlorophyll content, photosynthesis rates, soluble sugars, starch, and leaf soluble protein levels, while impairing antioxidant defense systems (Jain et al., 2011; Singh et al., 2025; Xu et al., 2025).

The application of calcium fertilizer has been shown to significantly improve the distribution ratios of nitrogen, phosphorus, potassium, and calcium in peanut plants, facilitating enhanced nutrient transport to pods and thereby increasing the number of mature pods per plant, pod dry weight, and kernel weight, which collectively contribute to improved yield (Yang et al., 2017; Li et al., 2024). Liu et al. (2021) demonstrated that calcium fertilization enhances photosynthetic rates, prolongs the peak activity of nitrogen metabolism-related enzymes, improves carbon assimilation capacity, and increases kernel yield, albeit with a suppressive effect on vegetative growth. Increasing calcium fertilizer application boosts photosynthetic product accumulation in upland peanuts by elevating the leaf net photosynthetic rate and delaying senescence, thereby significantly raising biomass and economic yield. Additionally, it enhances the activity of nitrogen metabolism enzymes throughout the growth cycle and carbon metabolism enzymes in early stages (Wang et al., 2015). Physiological analyses by Li et al., 2023 and Zuo et al. (2025) further revealed that calcium supplementation elevates antioxidant enzyme activity in leaves, reduces malondialdehyde (MDA) accumulation, and enhances overall physiological performance. Calcium nitrate application mitigates malondialdehyde (MDA) content and electrolyte leakage in peanut leaves while augmenting antioxidant enzyme activity (Huang et al., 2022).

The overuse of chemical fertilizers, reduced application of organic fertilizers, and widespread straw burning have also significantly exacerbated soil salinization and acidification. These processes have led to severe calcium depletion and markedly decreased available calcium content in soils (Abdissa et al., 2018. Adding quicklime to acidic soils not only adjusts soil pH but also enhances fertilizer use efficiency and crop yields (Gao et al., 2016; Liang et al., 2021). Desalegn et al. (2017) demonstrated that quicklime application effectively reduces soil acidity, alleviates aluminum and heavy metal toxicity, improves soil physical properties (e.g., aggregate stability, hydraulic conductivity), and increases soil calcium (Ca²^+^) contents. Furthermore, quicklime enhances dominant fungal abundance in continuously cropped peanut soils, with a greater impact on fungal community structure than peanut variety selection (Yang et al., 2024).

High-throughput RNA sequencing (RNA-seq) technology has been widely used to decipher the key molecular pathway and candidate genes involving Ca responses in different plant species. Through transcriptomic data indicated that carbohydrates, as important regulatory factors under drought stress, and key differential genes *SlGLR 3.2* and *SlGLR 3.5* affecting calcium transport in tomatoes under high vapor pressure deficit were identified (Shi and Du, 2023). In high calcium American ginseng, the core differential genes and core metabolites enriched in Glutamate metabolic pathway, compared with suitable calcium American ginseng, the gene (*Pg_S3572.10*) related with glutathione metabolism in high calcium American ginseng significantly up-regulated by 1.29 times (Zhang et al., 2022). Wang et al. (2022) study showed that DEGs of sweet potato responding to calcium stress were mainly enriched in plant cell wall, symplast and plasmodesma, and one candidate gene related to calcium metabolism was obtained by screening analysis. RNA-seq technology has been pod size (Lv et al., 2024), seed colours (Zhao et al., 2025), and disease (Wang et al., 2024) in peanut, but the mechanism of calcium on nitrogen remains to be systematically elucidated.

Peanut varieties exhibit substantial variation in calcium absorption and utilization efficiency, resulting in differential soil calcium requirements. Previous studies have predominantly focused on the effects of calcium application across different varieties under low-calcium stress, while research on the molecular mechanisms of calcium in nitrogen utilization among different peanut varieties remains scarce. With the completion of peanut genome sequencing, there has been gained a new research opportunity to analyze the characteristics of differentially expressed genes in different peanut varieties under calcium-sufficient and calcium-deficient conditions. This study employs RNA-seq transcriptome analysis to identify differentially expressed genes in different peanut varieties under calcium-sufficient and calcium-deficient conditions, aiming to achieve three main research objectives: (1) to investigate the effects of calcium on physiological characteristics, dry matter accumulation, and nitrogen utilization in different peanut varieties; (2) to identify calcium-responsive and nitrogen utilization-related genes through transcriptome analysis; and (3) to elucidate the new molecular mechanisms by which calcium promotes peanut growth and enhances nitrogen utilization.

## 2 Materials and methods

### 2.1 Field site

The two-year field experiment (2020-2021) was conducted in ZhengYang County, Henan Province, China (32°27′25″N, 114°20′28″E). The experimental site featured fluvo-aquic soil with sandy texture, characterized by the following properties: organic matter (11.9 g/kg), total nitrogen (112.3 mg/kg), available phosphorus (18.1 mg/kg), exchangeable calcium (80 mg/kg), available potassium (162.0 mg/kg), available molybdenum (65.0 μg/kg), available boron (120.1 μg/kg), and a pH of 5.2.

### 2.2 Experiment design

The experiment adopts a split-plot design, with the main plot being two treatment no Ca fertilizer (0) and increased calcium fertilization (CaO, 225 kg/hm^2^; Liang et al., 2021), while the subplot involves peanut varieties with different nitrogen utilization efficiency types. The low-Nitrogen-efficient peanut variety Puhua 66 and Shanhua 14, and high-Nitrogen-efficient peanut variety Puhua 28 and Huayu 20 were used for the experiments. Basal Ca application was performed 3 d prior to sowing. Using the autumn-sown wheat and summer-sown peanut cultivation mode with ridging, the ridge bottom width is 75 cm, the ridge surface width is 40 cm, and the ridge height is about 18 cm. Each plot contained four rows (row length, 10 m) of peanuts with an equal row spacing of 0.20 m. A randomized design with experimental plots at 3×10.0 m in size and 23,000 plants per ha in density was applied, with three replications of each treatment. Each plot was spaced 0.50 m from each other and used for sampling consisted of four central meters in two central rows. All plots received a banded basal application of 750 kg of compound fertilizer (N: P_2_O_5_: K_2_O=15:15:15) per ha, thereby maintaining the same amount of NPK fertilizer for each treatment. The application of quicklime in the field should be completed one month before peanut sowing. The specific procedure is as follows: uniformly spread the measured quicklime powder across the field, and immediately after spreading, it must be deeply incorporated using tools such as a rotary tiller to ensure thorough mixing with the 0-20 cm plow layer soil. Operators must wear masks, gloves, and protective goggles to prevent lime dust from causing burns to the skin, eyes, and respiratory tract. Standard agronomic management practices were implemented to control diseases, weeds, and insect pests.

### 2.3 Dry matter weight, nitrogen and calcium content in different organs of peanut

On the 45th day (flowering stage), 70th day (pod-setting stage), and 120th day (harvesting stage) after peanut sowing, five representative plant samples were collected from each plot and dissected into shoots, roots, and pods. Samples were oven-dried at 105 °C for 30 minutes followed by 75 °C until constant weight was achieved. We recorded total dry weight along with organ-specific (shoot, root, pod) dry weights. Dried samples were then ground to pass through an 80-mesh sieve for nutrient analysis. Nitrogen concentration was determined using a Kjeldahl nitrogen analyzer (UPT-K1800N) following H_2_SO_4_-H_2_O_2_ digestion, while calcium concentration was measured with a flame photometer (M410; Sherwood, UK) after HNO_3_-HClO_4_-HF digestion.

The calculation formulas for relevant indicators of N or Ca accumulation and utilization characteristics are as follows:

1. N or Ca accumulation in each organ of the plant (kg/hm^2^) = N or Ca content in that part of the plant × the dry matter weight of the part.
2. The proportion of N or Ca distribution in different organs (%) = the cumulative amount of N or Ca in the organ/the cumulative amount of nitrogen in the whole plant × 100%.
3. The absorption and translocation amount of N or Ca (mg/plant) = The cumulative amount of N or Ca in vegetative organs at the early podding stage - The cumulative amount of N or Ca in vegetative organs at maturity.
4. The translocation rate of N or Ca (%) = The absorption and translocation amount of N or Ca/The cumulative amount of N or Ca in vegetative organs at the early podding stage × 100%.
5. The contribution rate of N or Ca (%) = The translocation amount of N or Ca/The cumulative amount of N or Ca in pods × 100%.
6. The production efficiency of N or Ca (kg/kg) = Pod weight/Total N or Ca accumulation in the whole plant at harvest time.
7. The harvest index of N or Ca (%) = N or Ca accumulation in pods at harvest time/Total N or Ca accumulation in the whole plant × 100%.

### 2.4 Determination of enzymatic antioxidant activity and nitrogen assimilation activity in leaves

Two fresh samples (each collected in duplicate) of the second leaf from the main stem apex and root systems from the 0~20 cm topsoil layer of peanut plants at the pod-setting stage were collected and stored at -80 °C. For peroxidase (POD) activity analysis, 0.5 g of finely ground leaf tissue was suspended in 3.0 mL of pre-cooled 50 mM phosphate-buffered saline (PBS) and centrifuged at 12,000 r for 20 min at 4 °C. One enzyme unit (U) was defined as the amount of enzyme required to inhibit the reaction by 50% under assay conditions. POD activity was measured by adding 30 µL of leaf enzyme extract to a 3 mL reaction mixture (0.2 M PBS, 0.38% guaiacol, 0.17% H_2_O_2_), with absorbance monitored at 470 nm. Enzyme activity was expressed as Δabsorbance units per minute (U/min).

Malondialdehyde (MDA) content, a marker of lipid peroxidation, was quantified via thiobarbituric acid (TBA) colorimetry as described by Vuković et al. (2022). Frozen leaf powder (0.5 g) was homogenized in 5% (w/v) trichloroacetic acid (TCA) and centrifuged at 12,000 × g for 20 min at 4 °C. The reaction mixture, consisting of 1.5 mL tissue extract and 1.5 mL reagent (0.67% TBA in 5% TCA), was incubated for 30 min at 100 °C. After cooling on ice, absorbance of the chromogenic product was measured at 450 nm, 532 nm, and 600 nm. MDA content was calculated using the formula: MDA content = 6.45 × (A532 − A600) −0.56 × A450.

Nitrate reductase (NR) activity was determined as described by Lea et al. (2006). Leaves (0.5 g) were homogenized in 4 mL of extraction buffer containing 0.1 M HEPES–KOH (pH 7.5), 3% (w/v) polyvinylpyrrolidone (PVP), 1 mM EDTA, and 7 mM cysteine (Cys). The assay mixture comprised 50 mM HEPES–KOH (pH 7.5), 100 mM NADH, 5 mM KNO_3_, and either 2 mM EDTA or 6 mM MgCl_2_ in a total volume of 2 mL. NR activity was measured in crude extracts by quantifying nitrite (NO_2_^-^) formation after adding 1% (w/v) sulfanilamide and 0.2% (w/v) N-(1-naphthyl) ethylenediamine dihydrochloride in 3 M HCl. The activity state of NR was defined as the percentage of NR activity measured in the presence of Mg²^+^ (and 14-3-3 proteins) relative to that measured in the presence of EDTA, reflecting the proportion of the enzyme in its nonphosphorylated, active form. All assays were conducted at 25 °C.

Glutamine synthetase (GS) activity was determined in peanut leaves using the hydroximate biosynthetic method described by Pompelli et al. (2010). GS activity was measured with a UV–VIS spectrophotometer (UV-1650PC, Shimadzu, Kyoto, Japan) by quantifying the production of γ-glutamyl-hydroxamate at 540 nm.

### 2.5 Total RNA extraction, transcriptome sequencing, and analysis

No Ca fertilizer and increased calcium fertilization leaf from 4 peanut cultivar used for were the same as those used for RNA sequencing (RNA-Seq). Three independent biological replicates were taken for each cultivar for a total of 24 samples. RNA-seq library construction and subsequent analyses were performed by Biomarker Technologies (Beijing, China). Total RNA was extracted from peanut leaves during the pod-setting stage and evaluated for concentration, purity, and integrity using agarose gel electrophoresis, a Qubit 2.0 fluorometer, a NanoPhotometer spectrophotometer, and an Agilent 2100 Bioanalyzer. Samples meeting quality standards were retained for downstream experiments. Following RNA purification, library preparation was executed, and the resulting cDNA libraries were sequenced on the Illumina HiSeq 4000 platform. Raw reads generated were subjected to quality control (QC) procedures, followed by adapter trimming using SOAPfuse software to produce clean data. Clean reads were mapped to the reference genome of peanut cultivar Tifrunner (available at: www.Peanutbase.org) using HISAT2 v2.1.0. Transcript assembly was conducted with StringTie (accessed via http://ccb.jhu.edu/software/stringtie/on September 15, 2022). Gene expression levels were quantified using FPKM (Fragments Per Kilobase of transcript per Million mapped reads), calculated as:FPKM = (Number of cDNA fragments)/(Total mapped reads in millions) × (Transcript length in kilobases). Differentially expressed genes (DEGs) were identified based on a false discovery rate (FDR) < 0.001 and |log_2_(Fold Change)| ≥ 1. Functional annotation of DEGs was performed using KOBAS v2.1.1, incorporating Gene Ontology (GO; http://geneontology.org/) and Kyoto Encyclopedia of Genes and Genomes (KEGG; https://www.kegg.jp/) databases. Additionally, DEG sequences were aligned and annotated against the Nr, Swiss-Prot, GO, and KEGG databases using BLAST software to obtain comprehensive annotation information. Principal component analysis (PCA), Pearson correlation coefficient (PCC) calculations, and weighted gene co-expression network analysis (WGCNA) were executed on the BMKCloud platform (https://international.biocloud.net) to further characterize the transcriptome data.

### 2.6 Real-time quantitative PCR validation

RNA extraction was performed using TRIzol reagent (Invitrogen, Waltham, USA). Quantitative fluorescence primers were designed using Primer 5.0 software (**Supplementary Table S1**). Total RNA was used as the template for cDNA synthesis, which was performed using reverse transcriptase (Takara Bio, Dalian, China) according to the manufacturer’s instructions. For cDNA synthesis and quantitative real-time PCR (qRT-PCR), a previously published method was employed (Xu et al., 2022). The quality of the reverse-transcribed cDNA was verified using primers for the peanut internal control gene, actin.

### 2.7 Statistical analysis

The statistical analysis was performed using the DPS 7.5 software with Duncan’s test (*p* < 0.05). Using Microsoft Excel 2007 and Origin 8.5 software to organize data and create graphs.

## 3 Results and analysis

### 3.1 The impact of calcium fertilizer on the enzyme activity of peanut leaves

**Figure 1:**
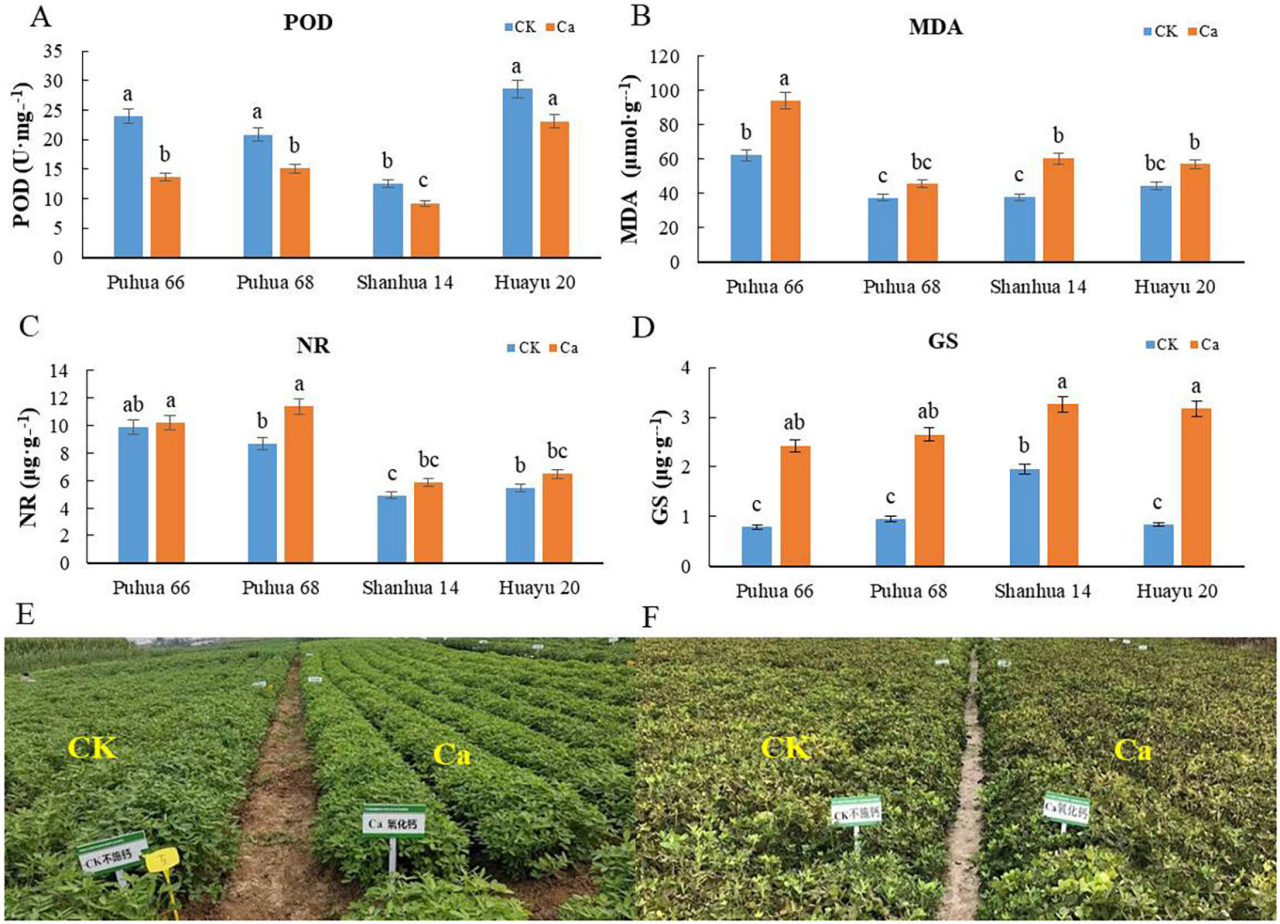
Effect of calcium fertilizer on enzyme activity of leaves and senescence performance of pod and maturity stage for peanut. **(A)** POD activity of peanut leaves; **(B)** MDA activity of peanut leaves; **(C)** NR activity of peanut leaves; **(D)** GS activity of peanut leaves; **(E)** Peanut senescence performance of pod stage; **(F)** Peanut senescence performance of maturity stage. Different capital letters indicate significant difference among treatments (*P* < 0.05). The same as blow.

Peroxidase and malondialdehyde are widely present in plants and play regulatory roles in plant development. As shown in **Figure 1**, the POD activity in peanut leaves decreased after the application of additional calcium fertilizer across all varieties, with significant reductions observed in Puhua 66, Puhua 28, and Shanhua 14. Conversely, MDA levels increased in the leaves of all varieties following calcium fertilizer application, with significant increases in Puhua 66 and Shanhua 14. Moreover, the application of additional calcium fertilizer resulted in more concentrated flowering across different peanut varieties, a shortened duration of functional leaves, and an accelerated completion of the entire life cycle (**Figures 1E, F**). These changes suggest that increased calcium fertilizer application enhances MDA activity, reduces POD activity, accelerates plant growth and development, and promotes early maturity in peanut plants. Nitrate reductase and glutamine synthetase are key enzymes in ammonium assimilation and nitrogen metabolism. As shown in **Figure 1**, the activities of both NR and GS in peanut leaves increased after the application of additional calcium fertilizer. Notably, GS activity reached significant levels in all varieties. This finding is consistent with T1, indicating that calcium fertilizer application promotes GS activity in the later stages of peanut growth. The T2 treatment exhibited higher GS activity across all four growth stages compared to other treatments, suggesting that increasing calcium fertilizer application while ensuring adequate nitrogen fertilizer enhances GS activity in peanuts.

### 3.2 The impact of calcium fertilizer on dry matter accumulation of peanuts

**Figure 2:**
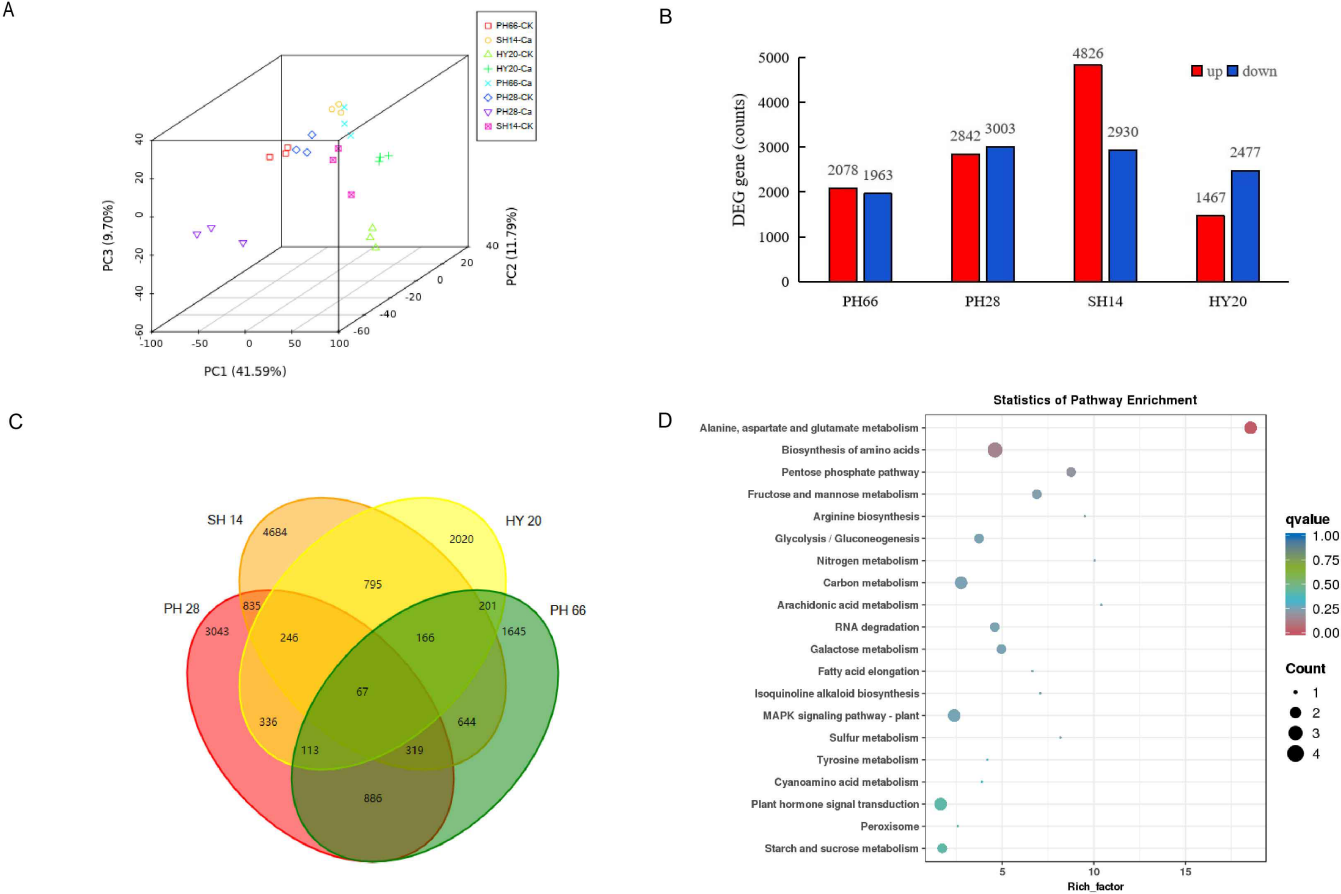
Effect of calcium fertilizer on different organs dry matter in different stage peanut. **(A)** Puhua 28 different organs dry matter; **(B)** Shanhua 14 different organs dry matter; **(C)** Puhua 66 different organs dry matter; **(D)** Huayu 20 different organs dry matter.

Calcium fertilizer treatments increased dry matter accumulation in peanut nutritional organs (shoots and roots) by 3.8% to 39.9% compared to the control (CK) without calcium fertilizer, while pod dry matter accumulation was significantly increased in all four peanut varieties (**Figure 2**). The accumulation trends of shoots, roots, and total dry matter under different calcium fertilization treatments were similar. During the seedling stage, calcium-treated plants exhibited lower total, shoot, and root dry matter accumulation than CK, particularly for shoot and total dry matter accumulation in Puhua 28 and Huayu 20, which reached significant levels. During the pod-setting stage, total and shoot dry matter accumulation were lower than CK, while root and pod dry matter accumulation were higher than CK. Specifically, pod dry matter accumulation increased by 26.9%, 10.6%, 67.2%, and 30.2% in Puhua 28, Puhua 66, Shanhua 14, and Huayu 20, respectively. At maturity, compared to CK, total dry matter accumulation increased by 1.1%, 6.5%, 9.4%, and 1.3% in Puhua 28, Puhua 66, Shanhua 14, and Huayu 20, respectively, while pod dry matter accumulation increased by 19.7%, 20.9%, 62.9%, and 48.3%, respectively. However, shoot dry matter accumulation decreased by 11.2%, 3.79%, 25.0%, and 39.0%, respectively. These results indicate that although calcium fertilizer reduces dry matter accumulation in the early growth stage, it promotes the translocation of dry matter from shoots to pods in the middle and late stages, ultimately increasing peanut yield (**Figure 2**).

### 3.3 The impact on peanut nitrogen absorption and utilization

Significant differences in nitrogen accumulation across different peanut organs are attributed to variety, additional calcium fertilizer application, and their interaction (**Table 1**). For the same variety, nitrogen accumulation in shoot dry matter is significantly reduced under additional calcium fertilizer, while pod nitrogen accumulation significantly increases, with minimal impact on roots. Among the same treatments, high-nitrogen varieties, such as Puhua 28 and Huayu 20, exhibit significantly higher pod nitrogen accumulation than low-nitrogen varieties, such as Puhua 66 and Shanhua 14. Compared to the control, calcium fertilizer treatment increases pod nitrogen accumulation by 89.13 g/kg, 143.11 g/kg, 307.03 g/kg, and 420.53 g/kg in Puhua 28, Puhua 66, Shanhua 14, and Huayu 20, respectively.

**Table 1:** Effects of different calcium treatments on nitrogen accumulation and distribution in peanut.

Varieties	Treatments	N accumulation (kg/hm^2^)			N distribution (%)			N accumulation amount (kg/hm^2^)	N translocation amount (g/kg)	N transport rate (%)	N harvest index	N production efficiency (kg/kg)
		Root	Shoot	Pod	Root	Shoot	Pod					
PH 28	CK	0.74a	6.98b	17.34ab	2.97a	27.84b	69.19bc	25.07ab	203.70a	31.44ab	0.69ab	16.62bc
	Ca	0.41c	6.24b	19.92a	1.53b	23.49bc	74.98a	26.56a	212.39a	36.52a	0.75a	18.96b
PH 66	CK	0.67ab	8.12a	12.20bc	3.21a	38.68a	58.11c	20.99c	115.03c	20.56c	0.58c	18.88b
	Ca	0.68ab	6.66b	13.80bc	3.23a	31.49ab	65.28bc	21.14c	138.03c	25.29b	0.65b	22.46a
SH 14	CK	0.35c	7.90ab	9.48c	1.99b	44.55a	53.46c	17.72c	168.89b	31.98ab	0.53c	15.98c
	Ca	0.48bc	5.05b	15.00b	2.33ab	24.60bc	73.07a	20.53c	179.49b	36.89a	0.73a	22.47b
HY 20	CK	0.51bc	7.33ab	15.57b	2.20ab	31.31ab	66.48b	23.4b	105.32c	25.00b	0.66b	14.78c
	Ca	0.72a	4.25c	23.14a	2.57ab	15.10c	82.32a	28.11a	133.41c	32.58a	0.82a	18.26b
	CK	0.57a	7.58a	13.65b	2.59a	35.60a	61.81b	21.80b	148.23b	27.25b	0.62b	16.57b
	Ca	0.57a	5.55b	17.96a	2.42a	23.67b	73.91a	24.09a	165.83c	32.82a	0.74a	20.54a
	PH 66	0.68a	7.39ab	12.99bc	3.22a	35.09a	61.69b	21.06c	126.52b	22.93b	0.62b	20.67a
	PH 28	0.58ab	6.61b	18.63ab	2.25b	25.66b	72.09a	25.82ab	208.05a	33.98a	0.72a	17.79b
	SH 14	0.42b	6.48b	12.24bc	2.16b	34.57a	63.27b	19.13bc	174.19ab	34.43a	0.63b	19.23a
	HY 20	0.62a	5.79c	19.35ab	2.39b	23.21b	74.40a	25.76a	119.36b	28.80ab	0.74a	16.52b
	Variation source											
	N rate	ns	*	*	ns	*	*	ns	*	*	*	*
	Varieties	*	*	*	*	*	*	*	*	*	*	*
	N × Varieties	*	*	*	*	*	*	*	*	*	*	*

Data are two-year means. Different letters and * indicates a significant correlation at the 0.05 level, and ** indicates a very significant correlation at the 0.01 level, the same below.

Distribution rates increase by 7.7%, 5.79%, 19.61%, and 15.84%, respectively, while nitrogen translocation rates rise by 4.73%, 5.08%, 4.91%, and 7.58%, respectively. Under the interaction of variety and calcium fertilizer application, Huayu 20 with additional calcium fertilizer achieves the highest total pod nitrogen accumulation, with a nitrogen distribution ratio of 82.82%. Calcium fertilizer application promotes nitrogen allocation to reproductive organs thereby increasing the nitrogen harvest index and nitrogen use efficiency. Compared to no calcium fertilizer application, the pod harvest index of Puhua 28, Puhua 66, Shanhua 14, and Huayu 20 significantly increases by 0.07, 0.06, 0.20, and 0.16, respectively, while nitrogen use efficiency increases by 3.58 kg/kg, 2.34 kg/kg, 6.49 kg/kg, and 3.48 kg/kg under calcium fertilizer treatments.

### 3.4 Absorption and utilization of calcium in peanuts

As shown in **Table 2**, calcium accumulation in the roots is significantly reduced under additional calcium fertilizer, while pod calcium accumulation significantly increases. The overall calcium distribution across various organs follows the order: pods > shoots > roots. Compared to no calcium fertilizer application, calcium accumulation in the pods of Puhua 28, Puhua 66, Shanhua 14, and Huayu 20 significantly were increased by 602.28 kg/hm², 351.00 kg/hm², 1186.38 kg/hm², and 1041.48 kg/hm², respectively. Total plant calcium accumulation significantly increased by 475.02 kg/hm², 415.44 kg/hm², 612.54 kg/hm², and 848.16 kg/hm², respectively, while root calcium accumulation decreased by 149.04 kg/hm², 160.56 kg/hm², 58.50 kg/hm², and 52.92 kg/hm² under calcium fertilizer treatments. Calcium fertilizer application not only was increases calcium accumulation in various organs but also significantly enhances calcium translocation rate, production efficiency, and harvest index. The calcium translocation rate of Puhua 28, Puhua 66, Shanhua 14, and Huayu 20 were increased by 6.97%, 17.64%, 31.56%, and 13.85%, respectively. Among different varieties, Huayu 20 had the highest calcium harvest index, followed by Shanhua 14, then Puhua 28, with Puhua 66 having the lowest. The improvement in calcium production efficiency ranged from 8.03 to 34.36 kg/kg, with greater increases observed in nitrogen-inefficient varieties (Puhua 66 and Shanhua 14) compared to nitrogen-efficient varieties (Puhua 28 and Huayu 20), reaching significant levels.

**Table 2:** Effects of different calcium treatments on calcium accumulation and distribution in peanut.

Varieties	Treatments	Ca accumulation (kg/hm^2^)			Ca distribution (%)			Ca accumulation amount (kg/hm^2^)	Ca translocation amount (g/kg)	Ca transport rate (%)	Ca harvest index	Ca production efficiency (kg/kg)
		Root	Shoot	Pod	Root	Shoot	Pod					
PH 28	CK	0.31a	0.55b	1.99c	10.94a	19.12bc	69.94bc	2.85b	31.23a	39.58a	0.70b	146.01ab
	Ca	0.15b	0.77ab	2.35b	4.64b	23.58b	71.79b	3.27a	30.39a	37.22a	0.72b	154.04a
PH 66	CK	0.31a	0.79ab	2.21b	9.36a	23.85b	66.79bc	3.31a	25.68a	29.60ab	0.67bc	119.69c
	Ca	0.16b	0.81ab	2.81a	4.96b	25.00b	70.04ab	3.79a	31.15a	36.57a	0.70b	146.22ab
SH 14	CK	0.14b	0.94a	1.62c	5.00b	34.74a	60.20c	2.70a	5.47c	8.40c	0.60c	105.06c
	Ca	0.08c	0.42c	2.81a	2.36c	12.74c	84.91a	3.31a	18.46b	39.96a	0.85a	139.42b
HY 20	CK	0.16b	0.56c	1.94bc	3.96c	21.56b	74.48b	2.65b	8.65c	19.01b	0.74b	133.24ab
	Ca	0.10c	0.37c	2.98a	3.02c	10.61c	86.37a	3.45a	12.77bc	32.86a	0.86a	148.92ab
	CK	0.23a	0.71a	1.94b	7.33a	24.82a	67.85b	2.86b	17.76b	24.15b	0.68b	126.00b
	Ca	0.12b	0.59b	2.60a	3.74b	17.98b	78.28a	3.32a	23.19a	36.65a	0.78a	147.15a
	PH 66	0.24a	0.80a	2.24b	7.16a	24.43a	68.41b	3.28a	28.42a	33.08a	0.68b	132.96b
	PH 28	0.23a	0.66ab	2.17bc	7.79a	21.35ab	70.86b	3.06b	30.81a	38.40a	0.71b	150.02a
	SH 14	0.11b	0.68ab	2.22bc	3.71b	23.74a	72.55ab	3.00b	11.96b	24.18b	0.73b	122.24b
	HY 20	0.13b	0.46c	2.46b	3.49b	16.08b	80.43a	3.02b	10.71b	25.94b	0.80a	141.08a
	Variation source											
	Ca rate	*	ns	*	*	*	*	*	*	*	*	*
	Varieties	*	*	*	*	*	*	*	*	*	*	*
	Ca × Varieties	*	*	*	*	*	*	*	*	*	*	*

### 3.5 Calcium fertilizer application and transcriptome analysis in peanut leaves

**Figure 3:**
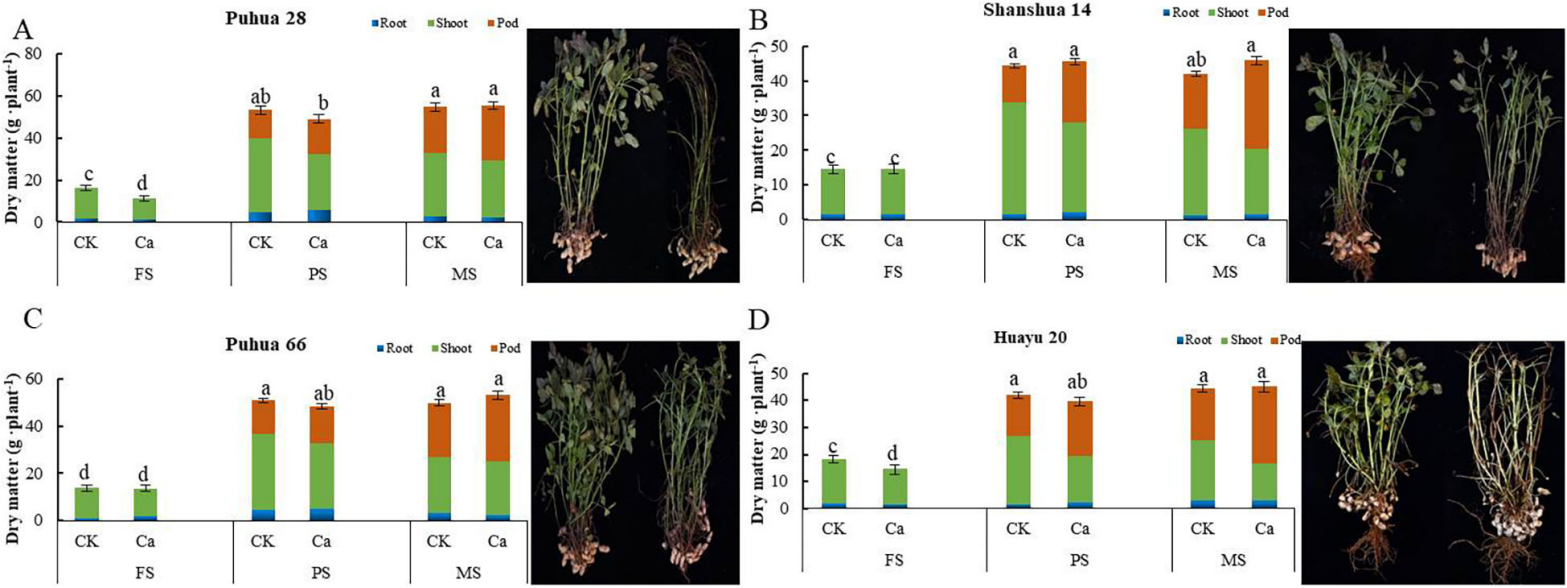
Transcriptome data of peanut accessions with calcium fertilizer application. **(A)** PCA analysis; **(B)** number of transcripts per varieties; **(C)** venn diagram number of up-regulated DEGs in different varieties; **(D)** DEG-enriched KEGG annotations.

To identify genes potentially involved in the response to calcium fertilizer, RNA-seq was performed on leaves from the pod-setting stage of different peanut varieties treated with additional calcium fertilizer compared to the control. Principal component analysis (PCA) and Pearson correlation coefficients revealed good repeatability among biological replicates, although the separation of the four varieties was not distinct, with correlation coefficients exceeding 0.9 (**Figures 3a**). In total, we obtained 21.17 million genes/transcripts and 137.5 Gb of clean reads from the 24 samples, averaging 5.7 Gb per sample. The Q30 values were 91.53% and 96.82%, with 96.82% of reads having quality scores at the Q20 level. Alignment efficiency ranged from 83.15% to 93.70%, yielding a total of 87,623 genes or transcripts. Among these, 66,667 genes were expressed in all four varieties (FPKM ≥ 0, FDR ≤ 0.001; **Figure 3c**). Pairwise comparisons identified 4,041 (Puhua 28), 7,756 (Puhua 66), 5,845 (Shanhua 14), and 3,944 (Huayu 20) differentially expressed genes (DEGs), with 3,086 overlapping DEGs. The criteria used to judge significant differences in gene expression (fold change > 2, FPKM ≥ 0.1, FDR ≤ 0.001) were stringent (**Figures 3b**). There were 166 genes specifically expressed in nitrogen-efficient varieties and 343 genes specific to nitrogen-inefficient varieties, with 67 overlapping DEGs (43 up-regulated and 24 down-regulated) (**Figure 3c**, **Supplementary Table S3**). And the 67 DEGS significant enrichment in KEGG pathways related to dry matter accumulation, nitrogen metabolism, early maturity (**Figure 3d**).

### 3.6 Identification of WGCNA modules associated with calcium fertilizer in peanut leaves

To further investigate key genes responsive to calcium fertilizer, we conducted weighted gene co-expression network analysis (WGCNA). A scale-free topological model was established using a soft threshold of 14. Using the dynamic hierarchical tree cutting algorithm, we identified 14 color modules (**Supplementary Figure S3a**). Among these, the purple module had the highest gene count (779 genes), while the white module had the lowest (34 genes) (**Supplementary Figure S3b**). Module-trait relationship analysis revealed that the black module exhibited significant correlations: a negative correlation with MDA and GS levels, and a positive correlation with POD activity (**Supplementary Figure S3d**). We then focused on the black module for functional enrichment analysis using GO and KEGG analyses. The results showed significant enrichment in KEGG pathways related to plant hormone signal transduction, ABC transporters, spliceosome, amino acid metabolism, starch and sucrose metabolism, and cell formation (**Supplementary Figure S4**).

**Figure 4:**
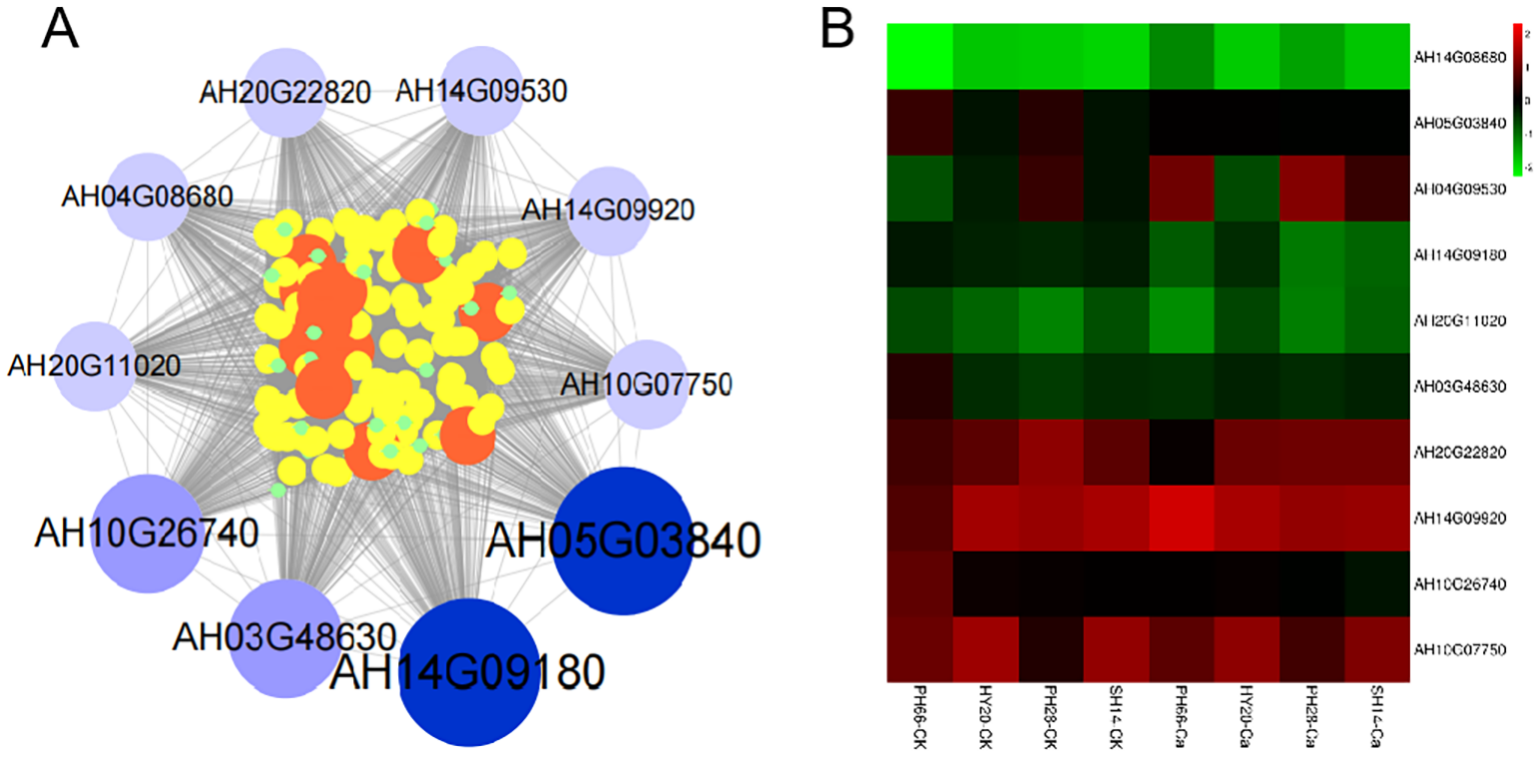
Hub genes based WGCNA. **(A)** Co-expression networks of the top 10 hub genes in the black; **(B)** Heatmap of the expression patterns of Hub genes.

A co-expression network was constructed to identify key genes involved in the response to calcium fertilizer. The top 10 hub genes with superior MCC scores were identified, and a visual gene interaction network diagram was generated for these genes and their associated genes (with a weight value greater than 0.20) (**Figure 4**). Functional annotation revealed that these 10 genes encode proteins involved in various biological processes: nucleic acid binding (AT-hook motif nuclear-localized protein 23, Remorin, C-terminal region), nitrogen metabolism (Stilbene synthase 3), growth and development (NDR1/HIN1-like protein 1, Chloroplastic import inner membrane translocase subunit), and other functions (Berberine bridge enzyme-like, NADH-ubiquinone reductase complex 1) (**Supplementary Table S5**). Under increased calcium fertilizer application, these core genes were down-regulated in the low-efficiency variety but up-regulated in the nitrogen-efficient variety.

### 3.7 Calcium improved expression of dry matter accumulation-related genes

**Figure 5:**
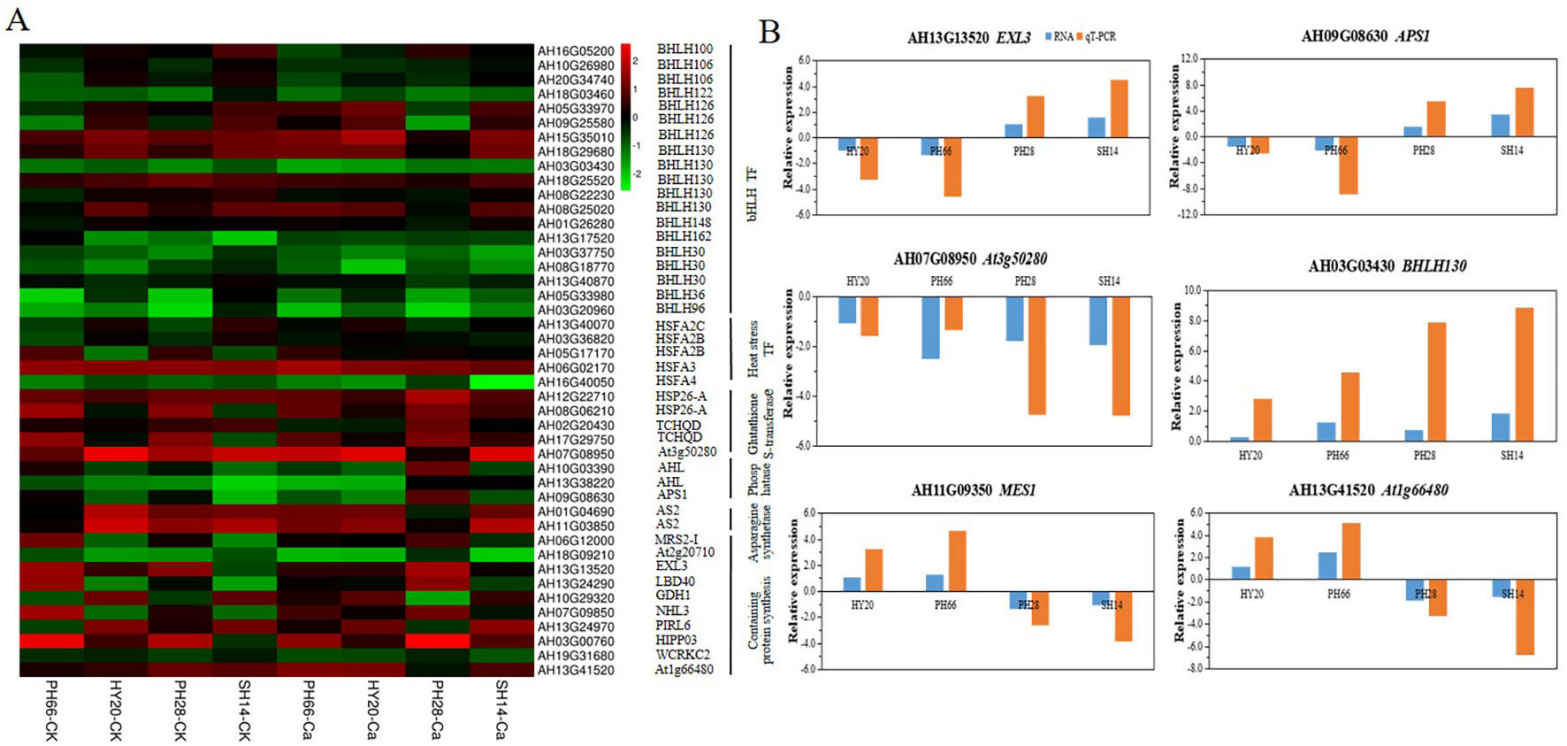
Expression differences of DEGs related to dry matter accumulation in black module based on WGCNA. **(A)** heatmap of the expression patterns of DEGs, FPKM values were normalized by Z-score; **(B)** qRT-PCR validation of key dry matter accumulation genes.

This study focused on the DEGs in the black module, as these genes play a crucial role in responding to calcium fertilizer application in peanut (**Figure 5a**). Through GO and KEGG analyses of the black module, terms related to dry matter accumulation, chlorophyll metabolism, and sugar metabolism were found to be enriched. A total of 31 DEGs associated with dry matter accumulation were identified. Under calcium fertilizer application, genes involved in calcium signaling (calmodulin, *HPT1*), photosynthesis (*PSAF, ISPF, NTH2, PSY, PCMP-H51*), and sugar synthesis (*AMY1.1*) were up-regulated, while genes involved in lipid metabolism (*At5g55050*), root phototropism (*RPT2*), and transport (*ABCC3, GAT1, ABCG14, UGT83A1*) were down-regulated. Notably, *ABCC3* was up-regulated in the nitrogen-efficient variety but down-regulated in the nitrogen-inefficient variety. Quantitative PCR (qPCR) results confirmed that the expression patterns of *ABCC3, UGT83A1, PSAF, AH13G38220, PSY*, and *At3g50280* were consistent with those obtained from RNA-Seq data (**Figure 5b**), validating the reliability of the RNA-Seq results.

### 3.8 Calcium improved expression of nitrogen metabolism-related genes

**Figure 6:**
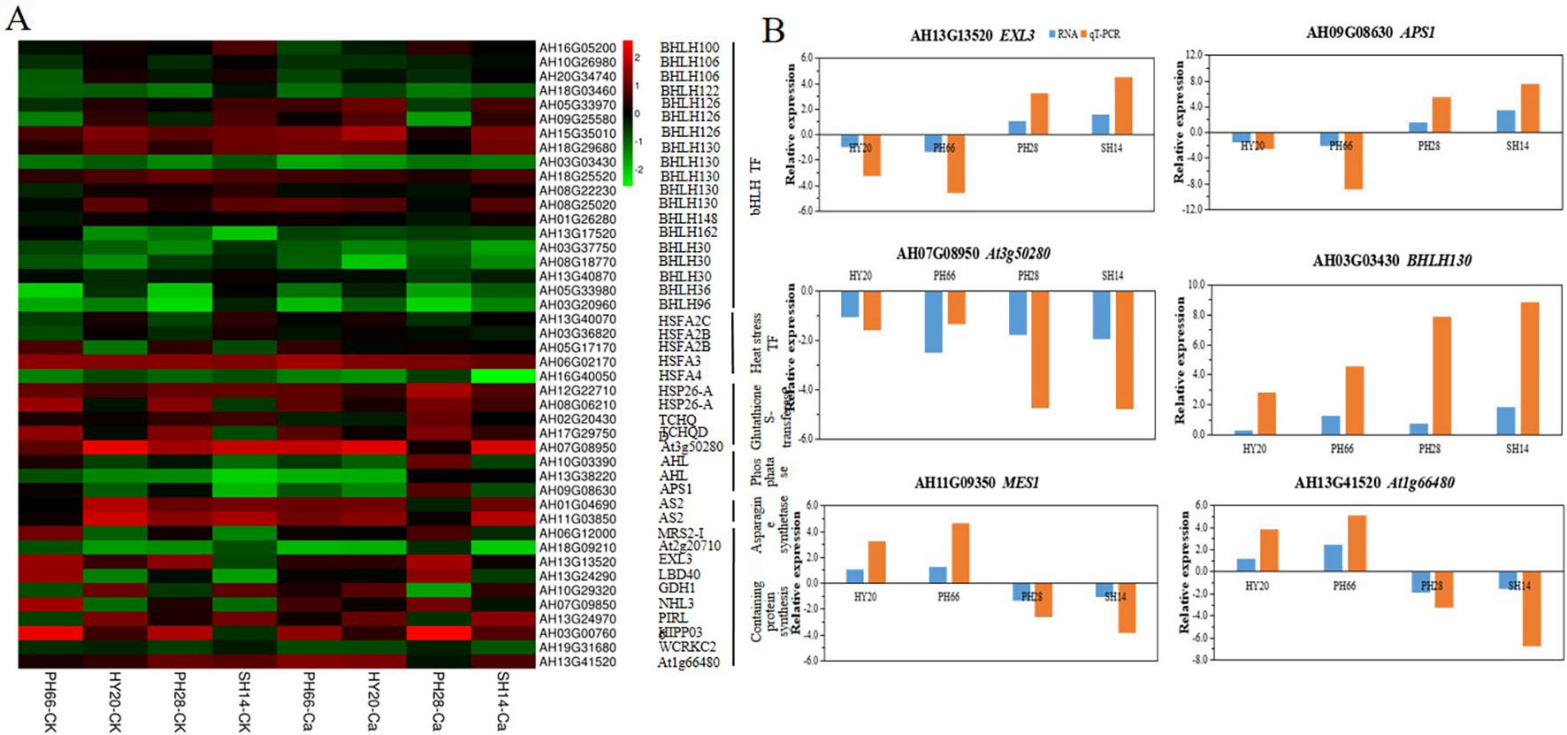
Expression differences of DEGs related to nitrogen metabolism in black module based on WGCNA. **(A)** heatmap of the expression patterns of DEGs, FPKM values were normalized by Z-score; **(B)** qRT-PCR validation of key nitrogen metabolism genes.

The black module of WGCNA identified 45 DEGs specifically associated with nitrogen metabolism and amino acid metabolism (**Figure 6a**). Under calcium fertilizer application, genes involved in amino acid binding (*CRK29, AS2, WCRKC2*), plant-pathogen interactions (*BHLH30, BHLH106, BHLH148, BHLH100, BHLH130*), and heat shock responses (*HSFA2C, HSP26-A*) were up-regulated, while genes involved in nitrogen metabolism (*NHL3*), domain-specific functions (*LBD40*), and magnesium ion transport (*MRS2-I*) were down-regulated. Additionally, *EXL3, CBP60B, MES1, APS1, BHLH126*, and *At1g66480* were up-regulated in nitrogen-efficient varieties but down-regulated in nitrogen-inefficient varieties. qRT-PCR analysis further confirmed that the expression trends of *EXL3, CBP60B, MES1, APS1, BHLH130*, and *At1g66480* were consistent with the RNA-seq results (**Figure 6b**), thereby demonstrating the reliability of the RNA-seq data.

### 3.9 Calcium improved expression of early maturity -related genes

**Figure 7:**
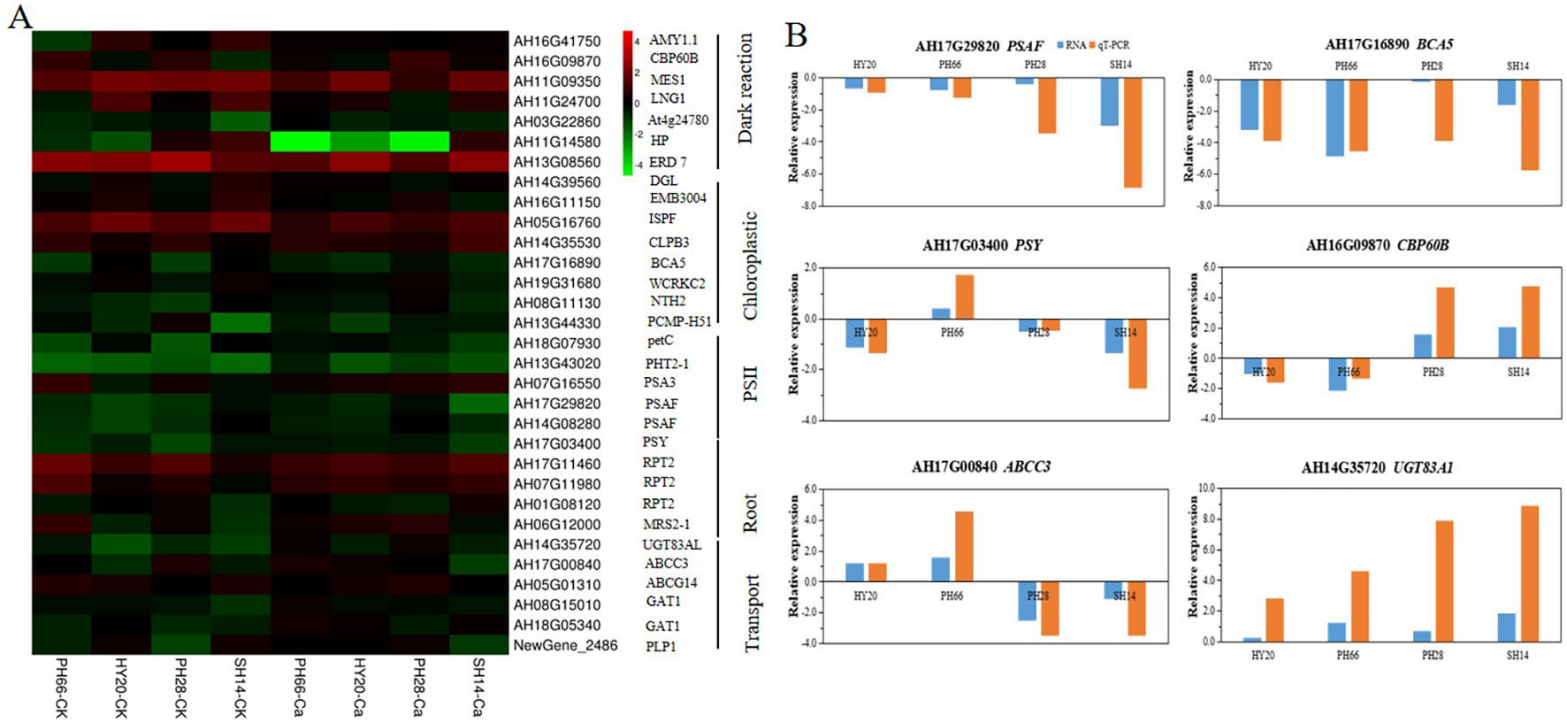
Expression differences of DEGs related to early maturity in black module based on WGCNA. **(A)** heatmap of the expression patterns of DEGs, FPKM values were normalized by Z-score; **(B)** qRT-PCR validation of key early maturity genes.

The black module from WGCNA identified 43 DEGs significantly enriched in terms related to programmed cell death, plant-pathogen interactions, kinases, and ethylene transcriptional synthesis (**Figure 7a**). Under increased calcium fertilization, genes related to ethylene (*ERF1B, ERF115, ERF110*), senescence (*MYB58, MYB86, MYB330*), plant circadian rhythm (*COL9*), and peroxidase activity (CSA) were up-regulated, while genes related to senescence (*ERF020, ERD7*), protein kinase activity (*RLP6*), and ethylene (*ERF020, ERF106*) were down-regulated. Additionally, the BTB/POZ domain protein (*At5g03250*), peroxidase (*PER20*), ethylene-related genes (*ERF1B, ERF011*), plant circadian rhythm gene (*At3g19360*), senescence-related genes (*MYB4, MYB3R5*), and receptor-like protein kinase (*RLP6*) were up-regulated in nitrogen-efficient varieties but down-regulated in nitrogen-inefficient varieties. qRT-PCR analysis confirmed that the expression trends of *FLZ6, MAKR5, ERF1B, MYB3R5, SAG20*, and *ASR2* were consistent with the RNA-seq results (**Figure 7b**), thereby demonstrating the reliability of the RNA-seq data.

In total, 14 genes related to plant precocity were identified, enriched in 16 pathways, including MAPK signaling pathway-plant (ko04016), plant hormone signal transduction (ko04075), plant-pathogen interaction (ko04626), biosynthesis of amino acids (ko01230), glycolysis/gluconeogenesis (ko00010), and protein processing in the endoplasmic reticulum (ko00030). Based on functional annotations, five of the DEGs encode ethylene-responsive transcription factors, while one DEG encodes the transcription factor MYB3R-5, which is involved in plant hormone signal transduction. Additionally, five other differentially expressed genes encode probable disease resistance proteins, peroxidase 20, abscisic stress-ripening protein, class I heat shock protein, and serine/threonine-protein phosphatase 7 long form homolog (**Supplementary Table S6**).

## 4 Discussion

### 4.1 Calcium improved dry matter accumulation and distribution of peanut pods

Dry matter accumulation is a key indicator of crop population quality. Typically, the distribution of dry matter among different plant parts determines peanut yield levels (Yang et al., 2020). After pod formation, the proportion of dry matter in leaves, stems, and petioles begins to decline, while the proportion in pods increases as they become the primary growth center. By maturity, the proportion of dry matter in pods reaches its peak (Wu et al., 2020). Soil acidification and saline-alkali stress can significantly inhibit dry matter accumulation in peanuts, while calcium deficiency severely impairs pod development. Calcium deficiency in the soil hinders the transport of photosynthetic products to pods, leading to poor pod development or abortion (Bi et al., 2024). Moreover, when photosynthetic products are retained in shoot organs, more resources are diverted to vegetative growth, potentially disrupting the balance between vegetative and reproductive growth and resulting in excessive vegetative growth. Calcium fertilizer application has no significant effect on the weight of vegetative organs but significantly (*p* < 0.0001) increases pod biomass and harvest index in red soil dryland (Wu et al., 2020). This study demonstrates that calcium application significantly enhances pod and whole-plant biomass. Additionally, nitrogen-efficient varieties exhibit greater increases in various indicators compared to nitrogen-inefficient varieties, with reproductive organs (pods) often showing higher increases than vegetative organs (stems, leaves, and roots). Calcium application also reduces the V/R (vegetative organs/reproductive organs) ratio, consistent with findings reported by Yang et al. (2017).

Further transcriptome sequencing analysis identified 31 genes associated with dry matter accumulation. These genes showed enrichment in pathways such as Photosynthesis (ko00195), carbon metabolism (ko01200), Starch and sucrose metabolism (ko00500), Pentose and glucuronate interconversions (ko00040), starch and sucrose metabolism (ko00500), and ABC transporters (ko02010) (**Supplementary Table S6**). Based on functional annotations, these differentially expressed genes encode TMV resistance proteins, ABC transporters, alkylamine oxidase, glutamate dehydrogenase, and aspartate synthase. Among them, *AH09G22950* and *AH19G28970* encode ethylene-responsive transcription factor 106 (*ERF106*), a member of the largest subfamily of ethylene-responsive factors (*ERF*s). Previous studies have shown that ERFs are key regulatory factors in plant development and stress responses (Xie et al., 2019). Specifically, the *APETALA2/ERF* subfamily is implicated in growth and developmental processes mediated by plant hormones such as gibberellins, cytokinins, and brassinosteroids in Arabidopsis. Another gene, *AH17G00840*, encodes an ABC transporter family protein. ABC transporters (ABCC and ABCG) have been shown to facilitate the transport of secondary metabolites, such as capsaicin and dihydrocapsaicin, into ovule vacuoles during pepper fruit ripening, thereby affecting their content and promoting fruit development (Lopez-Ortiz et al., 2019). Quantitative analysis indicated that genes associated with dry matter accumulation were up regulated under calcium fertilizer application. This suggests that these genes play a major role in regulating ABC transport and sugar metabolism pathways, thereby promoting dry matter accumulation and translocation towards the pods.

### 4.2 Calcium application on the accumulation and utilization of nitrogen and calcium in peanuts

Previous studies have shown that calcium nitrate application increases nitrogen accumulation and promotes its transport from vegetative organs to “sink” tissues in peanuts (Sadras et al., 2019). As calcium application increases, nitrogen and phosphorus accumulation and distribution rates in pods rise, while these rates in leaves, stems, roots, pegs, and shells significantly decrease (Bedoussac et al., 2014). Calcium application may positively affect nutrient absorption and accumulation in peanut organs under salt stress (Li et al., 2024). In this study, calcium fertilizer application significantly increased nitrogen content and distribution rates in roots and pods (underground organs) compared to the control without calcium treatment, while nitrogen content in stems and leaves (aboveground organs) and nitrogen distribution rates in leaves were significantly lower. These findings are consistent with changes in dry matter and nitrogen accumulation in peanuts, indicating that calcium application enhances nitrate reductase and glutamine synthetase activities, thereby increasing nitrogen absorption and utilization and providing a foundation for nitrogen accumulation. Nitrogen-efficient peanut varieties (Puhua 28 and Huayu 20) exhibited significantly higher nitrogen accumulation and distribution rates in pods compared to nitrogen-inefficient varieties. Transcriptome analysis revealed that 45 DEGs were enriched in three nitrogen metabolism pathways (**Supplementary Table S6**). Four of these genes were significantly enriched in the amino acid biosynthesis pathway (ko01230), with two genes (*AH09G22950, AH19G28970*) involved in converting fructose-6P to glutamate-P and the other two genes (*AH01G04690, AH11G03850*) involved in aspartic acid synthesis. Four genes were significantly enriched in the alanine, aspartate, and glutamate metabolism pathway (ko00250), with two genes (*AH01G04690, AH11G03850*) involved in L-asparagine synthesis and the third gene (*AH10G29320, AH03G20960*) involved in glutamate synthesis from NH_3_. These results suggest that calcium fertilizer application enhances NR and GS activities in leaves and directs more nitrogen to reproductive organs, preventing excessive vegetative growth and laying a foundation for improved plant population quality.

In plants, calcium primarily moves from the xylem to leaves via transpiration, which is the main driving force for calcium transport (Rahman et al., 2015). Calcium uptake in leaves under high calcium supply affects the absorption of other cations and influences stomatal morphology and photosynthesis (Johnson et al., 2022; Ma et al., 2022). Stomatal conductance, size, and density influence CO_2_ absorption, while CO_2_ diffusion and fixation depend on mesophyll tissue and chloroplast distribution (Tomeo and Rosenthal, 2017). CO_2_ diffusion involves passage through cell walls, plasma membranes, cytosol, chloroplast membranes, intercellular spaces, chloroplast matrix, and cell membranes in mesophyll cells (Du et al., 2019). Fruit hardness is affected by pectin and total calcium concentrations, which in turn affect cell wall structural stability (Althiab-Almasaud et al., 2021). Transcriptome analysis showed that major metabolic pathways involved in pod formation include phenylpropanoid metabolism, followed by starch, sugar, and glutathione metabolism, with lignin synthesis being a key factor. *AH05G03640* encodes a peroxidase involved in plant stimulus metabolism, transport, and catabolism, and participates in the synthesis of various lignin types in the phenylpropanoid biosynthesis pathway (ko00940) (**Supplementary Figure S5**). *NewGene_16049* is a novel gene involved in coenzyme transport and metabolism in the RNA transport pathway (ko03013) (**Supplementary Figure S5**). Calcium application increases calcium content in stems, roots, pegs, pods, and kernels, and raises calcium distribution rates in peanut leaves while decreasing rates in stems. These changes in photosynthetic product transport capacity, cell wall structural stability, carbohydrate and amino acid levels, and differentially expressed genes effectively improve calcium distribution and single pod weight under calcium application.

### 4.3 The effect of calcium application on the early maturity of peanuts

Plants rely on leaves to provide energy and exchange materials with the environment. As the growth period progresses, the active oxygen scavenging mechanism in peanut functional leaves becomes imbalanced. Calcium application enhances this mechanism, increasing chlorophyll content and the activities of SOD, POD, and catalase enzymes in peanut leaves, while decreasing MDA content. This helps eliminate or avoid damage caused by the accumulation of oxygen free radicals in leaves, which is crucial for normal plant growth (Jawad et al., 2020). MDA, a final product of lipid peroxidation, reflects the degree of reactive oxygen species (ROS) accumulation and damage to cell membranes under environmental stress (Huang et al., 2022). In recent years, the role of calcium has been widely discussed. Calcium ions (Ca²^+^) can directly or indirectly mediate various cellular signaling processes through calmodulin (CaM), modulating ion channel activities, gene expression, and oxidative stress tolerance. Calcium deficiency can disrupt the antioxidant protection system in peanuts, reducing CAT and POD activities, increasing MDA content, relative electrical conductivity, and superoxide radical (O_2_^-^) production rates, leading to increased cell membrane permeability and accelerated peanut senescence (Singh et al., 2025; Xu et al., 2025). Calcium application can effectively reduce peanut flowering time, promoting early flowering and facilitating the transition from vegetative to reproductive growth.

This study found that calcium application increased SOD, POD, and CAT activities and decreased MDA content in leaves of most peanut varieties, reducing cell membrane damage and cell sap exudation. This delays leaf aging, improves photosynthesis, and stabilizes cell structure and function, thereby increasing plant yields. Additionally, nitrogen-efficient peanut varieties (Huayu 20 and Puhua 66) showed more significant effects compared to nitrogen-inefficient varieties (Puhua 28 and Shanhua 14) under calcium fertilizer application. Transcriptome analysis annotated 45 DEGs with GO terms related to oxygen species metabolic processes. Five DEGs encoded ethylene-insensitive (EIN) proteins involved in pathway ko04626 (**Supplementary Table S6**). EIN is a major regulatory factor in the ethylene signaling pathway. For example, enhanced expression of *EIN3* promotes senescence and regulates ethylene-induced chlorophyll degradation in *Arabidopsis thaliana* leaves (Wang et al., 2020). *EIN3* also activates leaf senescence under adverse environmental conditions in Sorghum bicolor. Besides its role in leaf senescence, *EIN3* family genes are increasingly recognized for their involvement in defense responses to pathogens and environmental stimuli (Xu et al., 2023). For instance, overexpression of *EIN3* in *Arabidopsis* reduces resistance to Pseudomonas syringae, while suppressing this gene or in *EIL1* mutants enhances resistance, indicating a negative regulatory role in disease resistance (Wu et al., 2016). Similarly, *TaEIL1*, a homologue of *AtEIN3* from wheat, negatively regulates defense against wheat stripe rust (Duan et al., 2013). In this study, we observed up-regulation of *EIN* expression in peanut leaves, potentially leading to down-regulation of pathogen defense response transcripts. We found that calcium application in peanut fields led to preferential leaf shedding, earlier fruit development, and a shortened growth period, aligning with our hypothesis.

## 5 Conclusions

**Figure 8:**
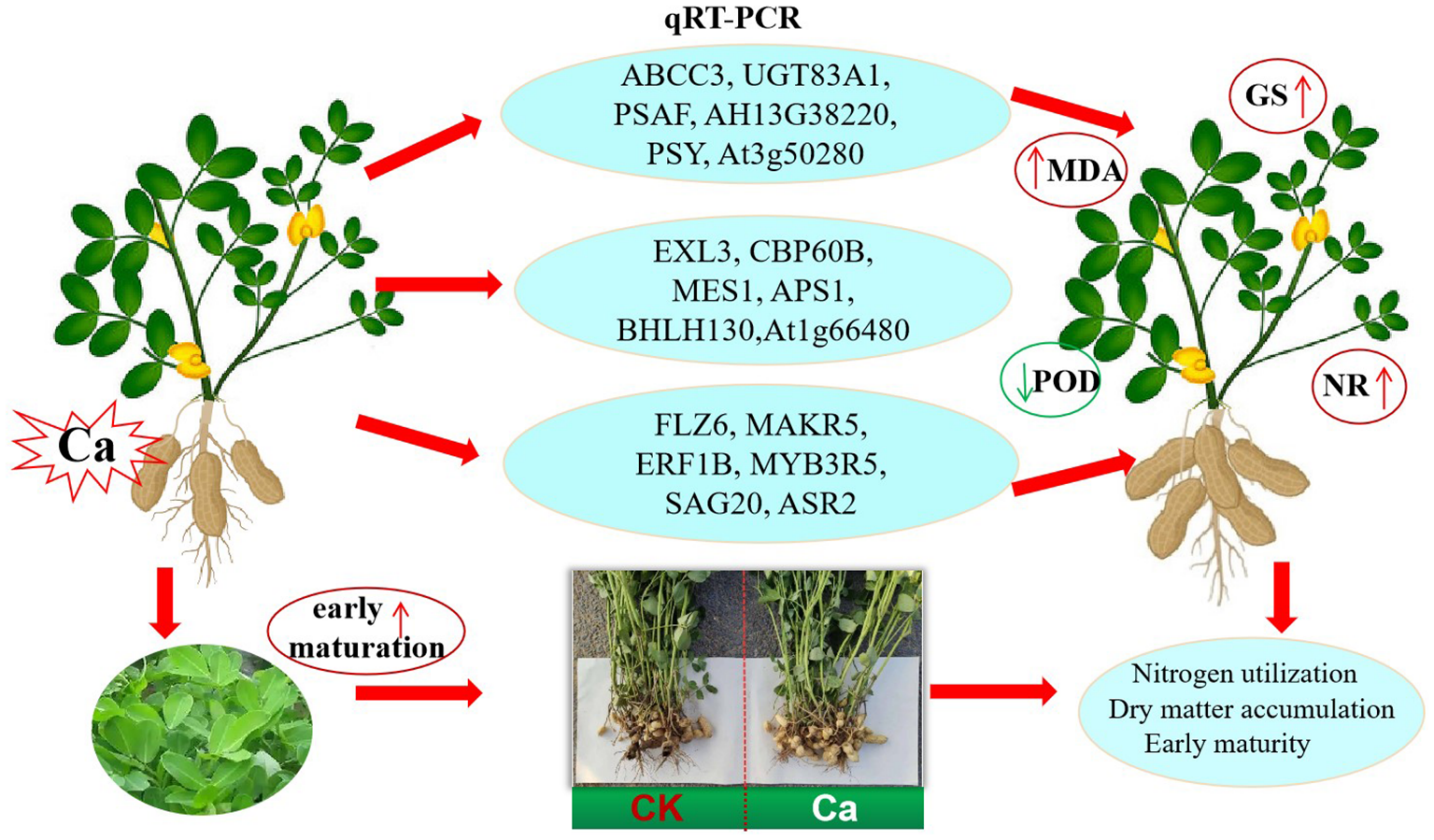
A molecular regulatory model for calcium application in peanut.

In summary, we sequenced the transcriptomes of peanut varieties treated with exogenous calcium. The DEGs were significantly enriched in carbohydrate metabolic processes, nitrogen metabolic processes, oxidoreduction processes, and photosynthetic processes. These DEGs were up-regulated under exogenous calcium treatment, leading to increased levels of GS, MDA, and NR in leaves. This enhancement significantly improved nitrogen use efficiency and the pod allocation ratio, as well as promoted plant maturity and yellowing, resulting in a shorter growth growth period for peanuts (**Figure 8**). This study investigates the effects of calcium application on the molecular and physiological aspects of peanut growth and development, providing scientific evidence that calcium fertilizer application can increase peanut yield in acidic or calcium-deficient soils.

## Data Availability

The data presented in the study are deposited in the NCBI, accession number PRJNA1314981.

## Supplementary material

Supplementary Table 1 Primer sequences used for quantitative real-time RT-PCR (qRT-PCR).

Supplementary Table 2 RNA-Seq reads quality control result parameters.

Supplementary Table 3 Expressions and annotation of DEGs in four comparable comple.

Supplementary Table 4 KEGG pathways significantly enriched by 4-coexpression DEGs.

Supplementary Table 5 Hub genes annotation.

Supplementary Figure 1 GO and KEGG enrichment specifically in low nitrogen efficiency varieties.

Supplementary Figure 2 GO and KEGG enrichment specifically in high nitrogen efficiency varieties.

Supplementary Figure 3 WGCNA process.

Supplementary Figure 4 KEGG and GO enrichment black module by WGCNA.

Supplementary Figure 5 The key pathways Ca for peanut grow.
