# Supplementary material for: Physiological and transcriptomic analyses of exogenous calcium in boosting nitrogen use efficiency via oxidative and resistance pathways in peanuts
Source: Front Plant Sci. 2026 Jan 27;16:1629610. doi: 10.3389/fpls.2025.1629610 (PMC12886391; doi:10.3389/fpls.2025.1629610)
Supplement: Supplementary Figure 1 — GO and KEGG enrichment specifically in low nitrogen efficiency varieties. [file DataSheet1.doc]

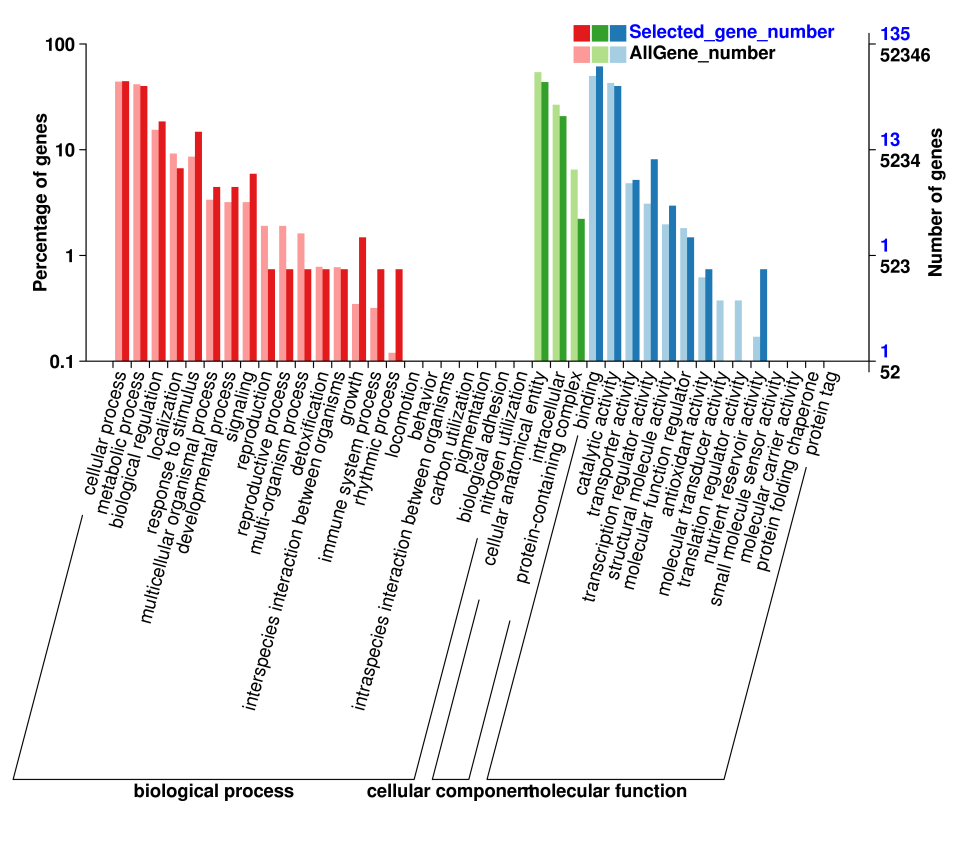

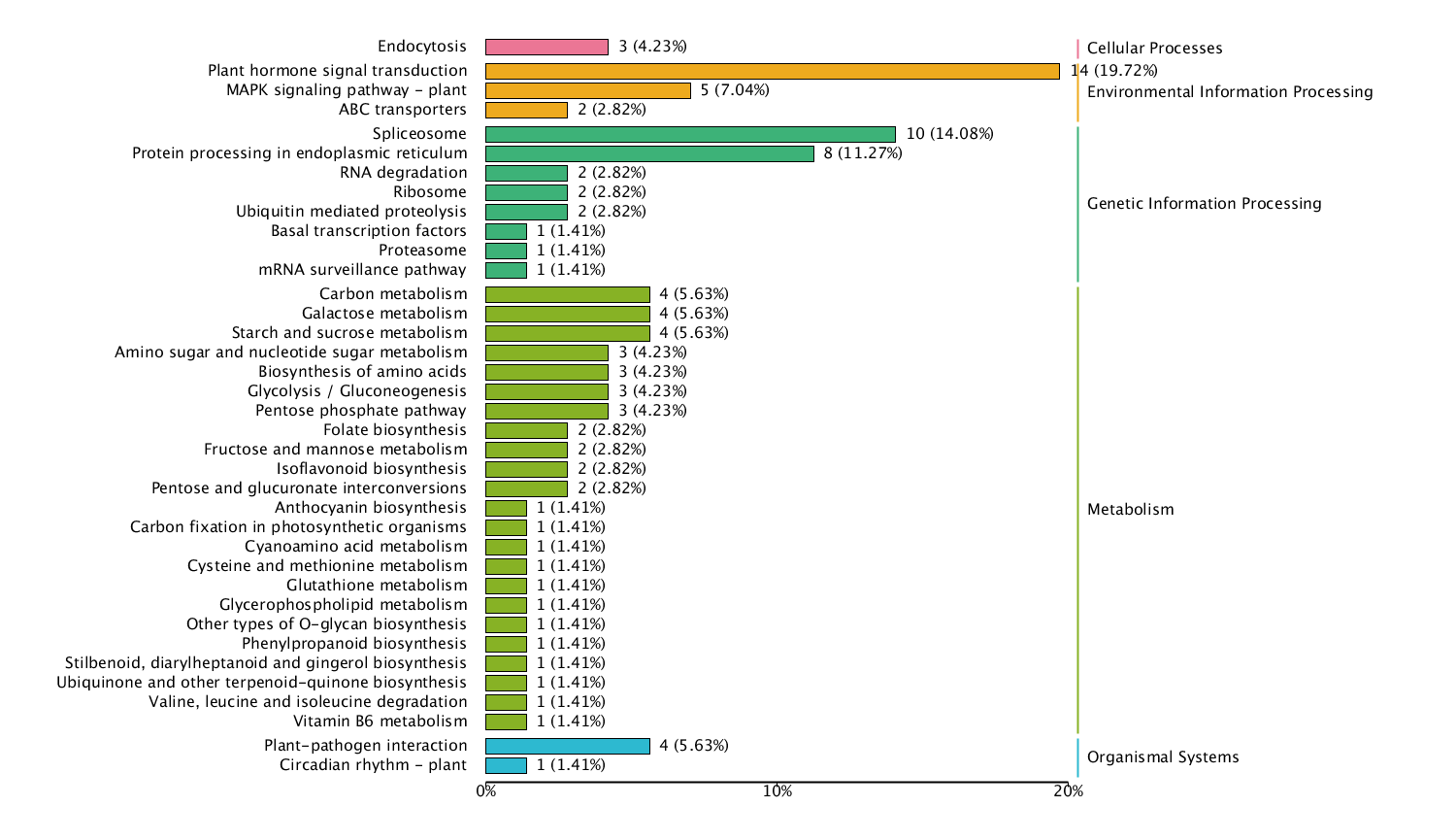


Figure S1. GO (Up) and KEGG (Down) enrichment specifically in low nitrogen efficiency varieties


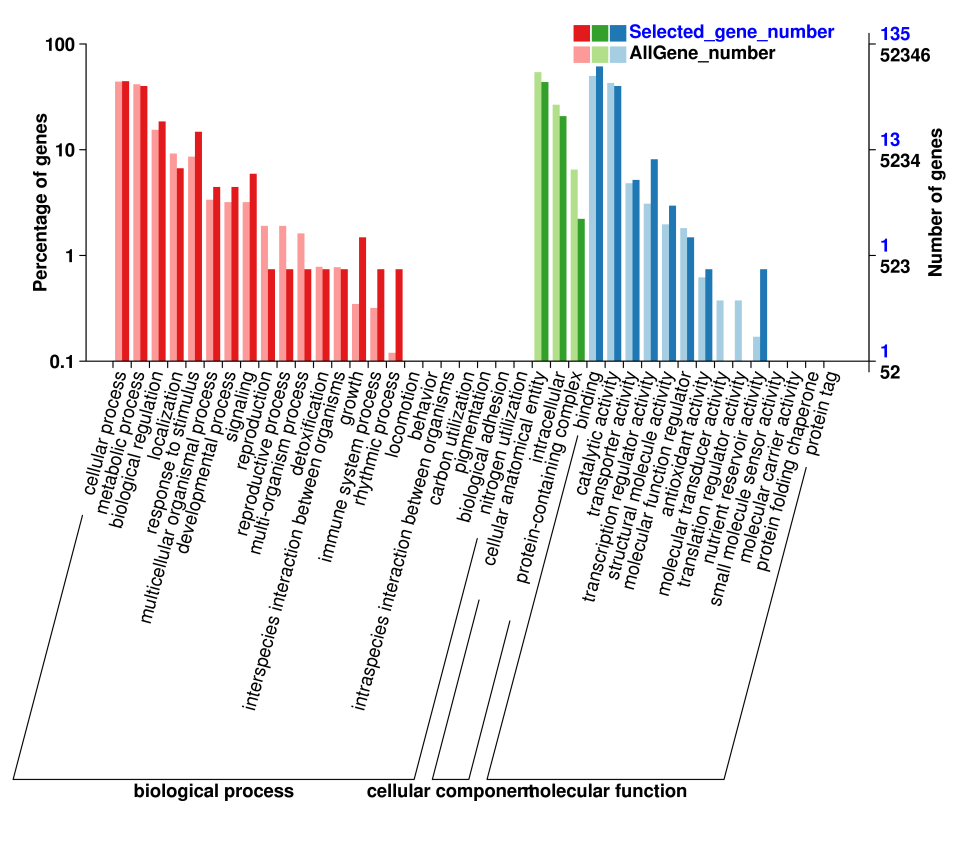

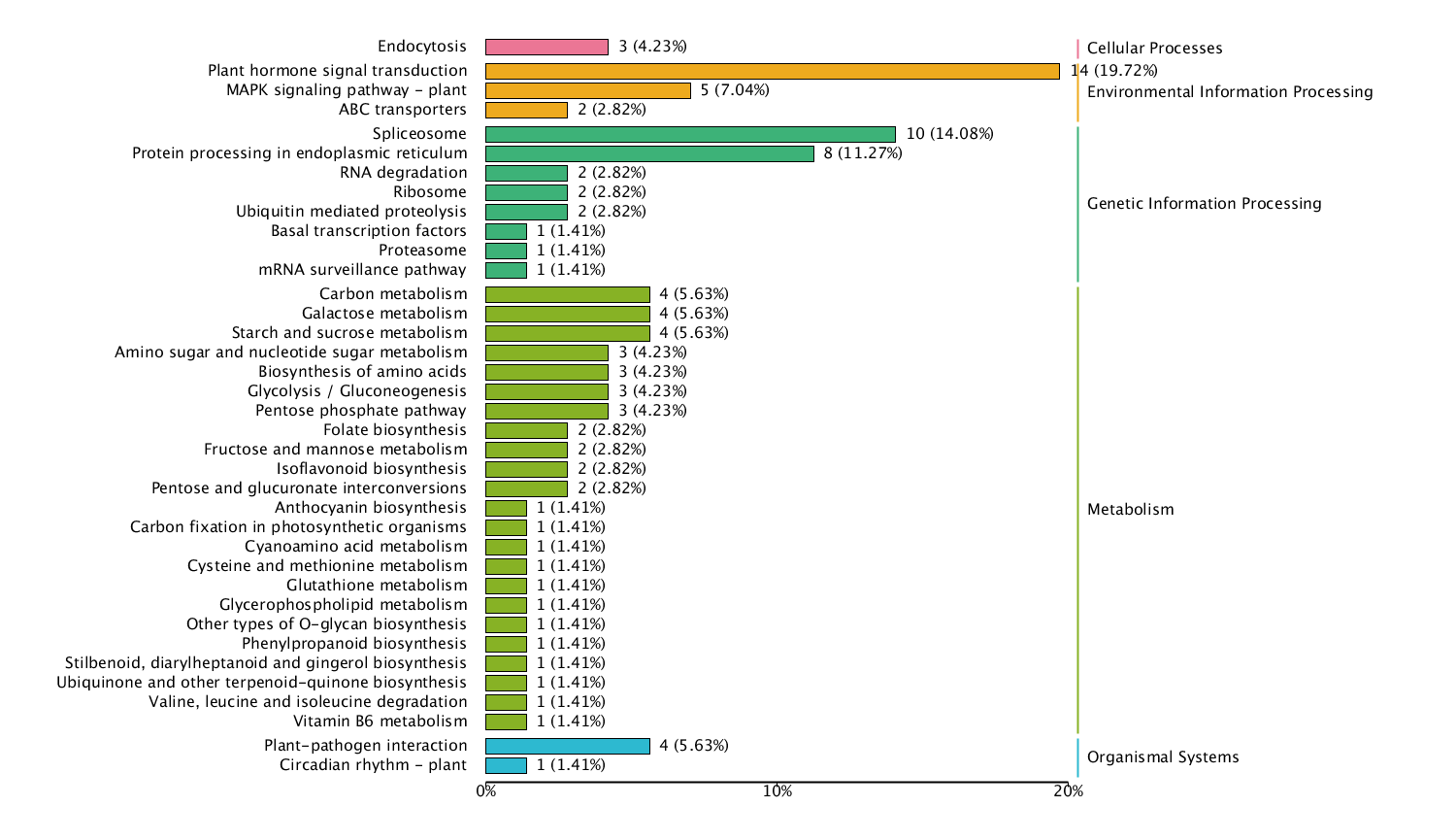


Figure S2. GO (Up) and KEGG (Down) enrichment specifically in high nitrogen efficiency varieties


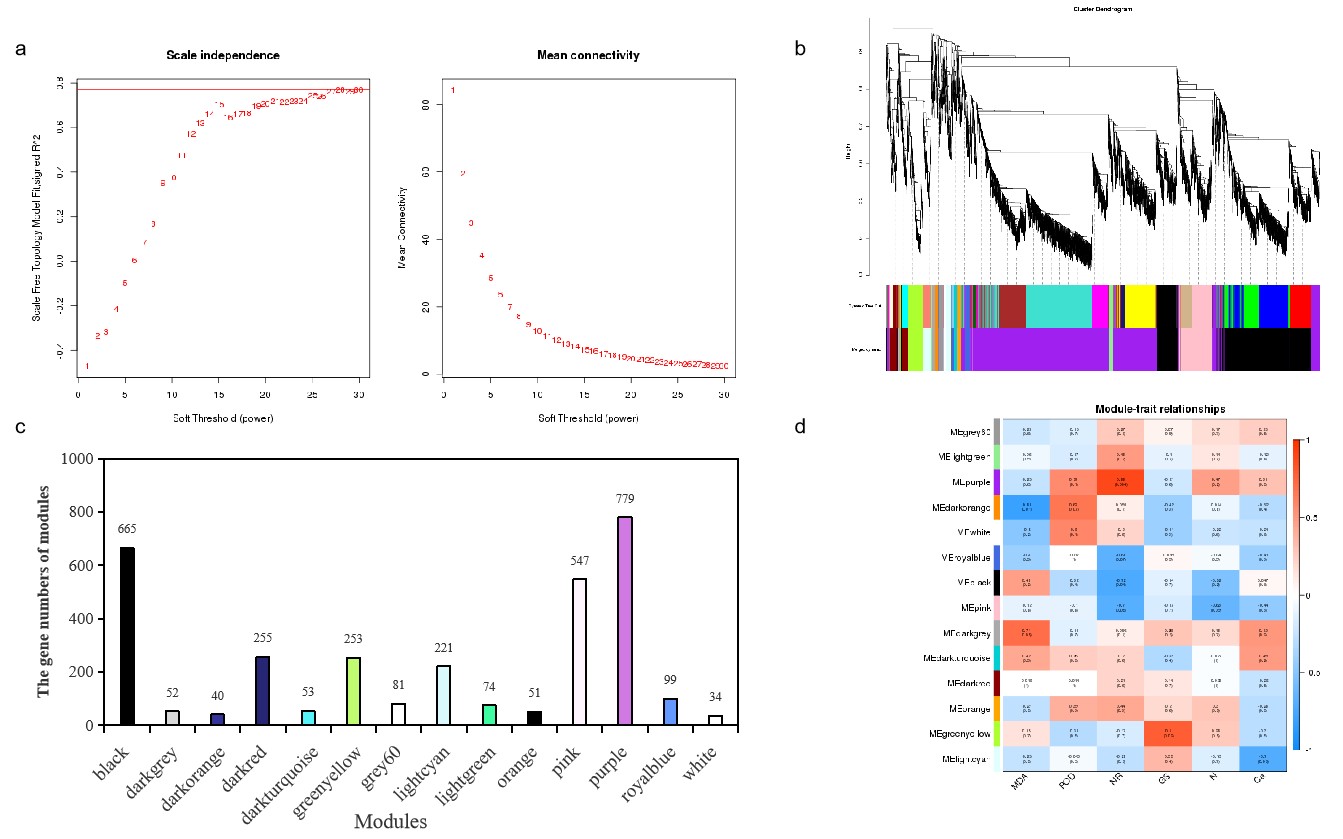


Figure S3. WGCNA process; a, network topology analysis of various soft thresholds. The left panel shows the scale-free fitting index (Y-axis) as a function of the soft threshold (X-axis). The right panel shows average connectivity (Y-axis) as a soft threshold (X-axis); b, systematic clustering tree of gene, gene network/module generated by dynamic shear method and gene network/module after merger; c, the genes number of module; d, relationships between modules and traits. The darker colors indicate higher correlation coefficients. Numbers represent Pearson’s correlation coefficients R^2^-values and the p-value for the correlation


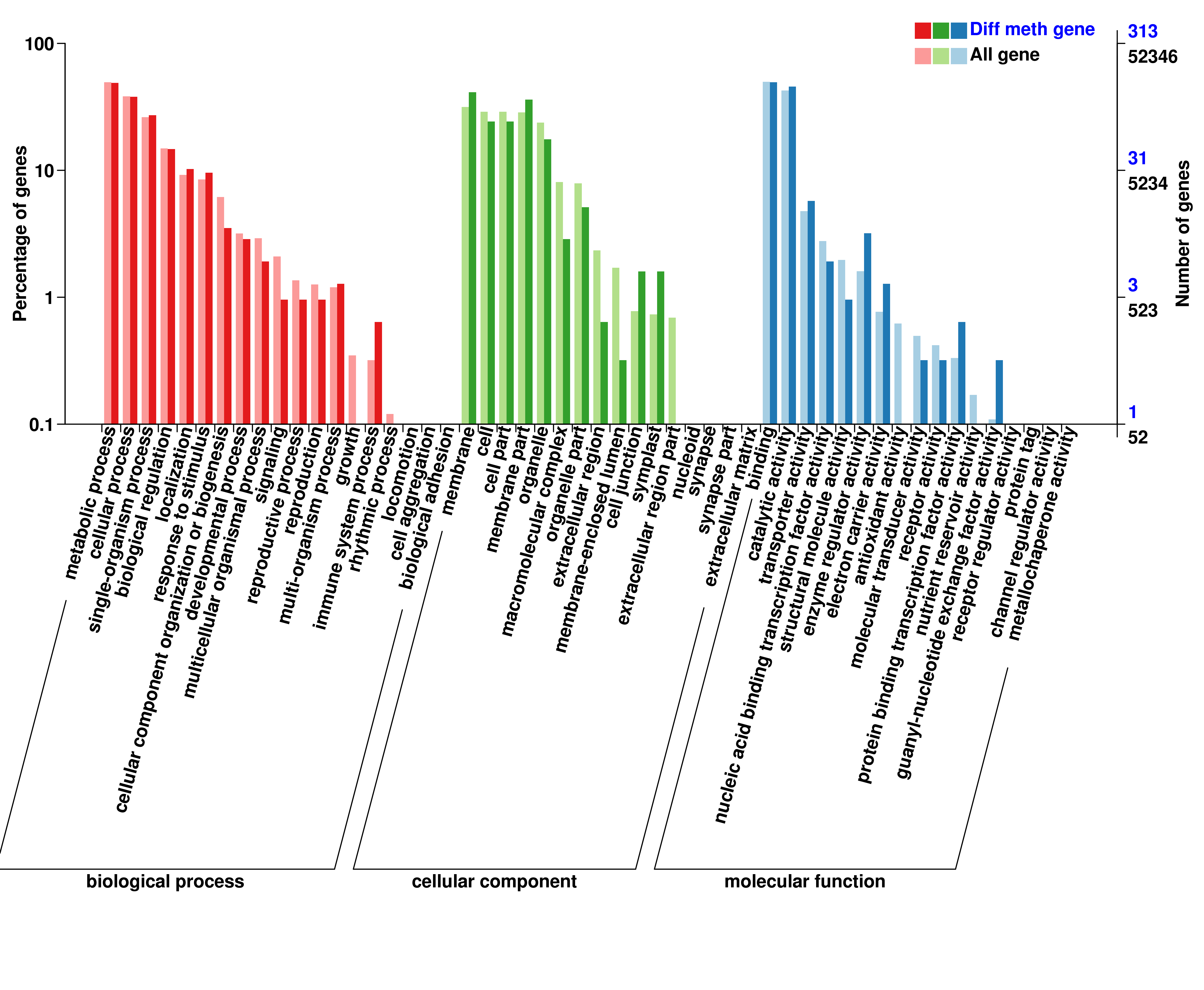

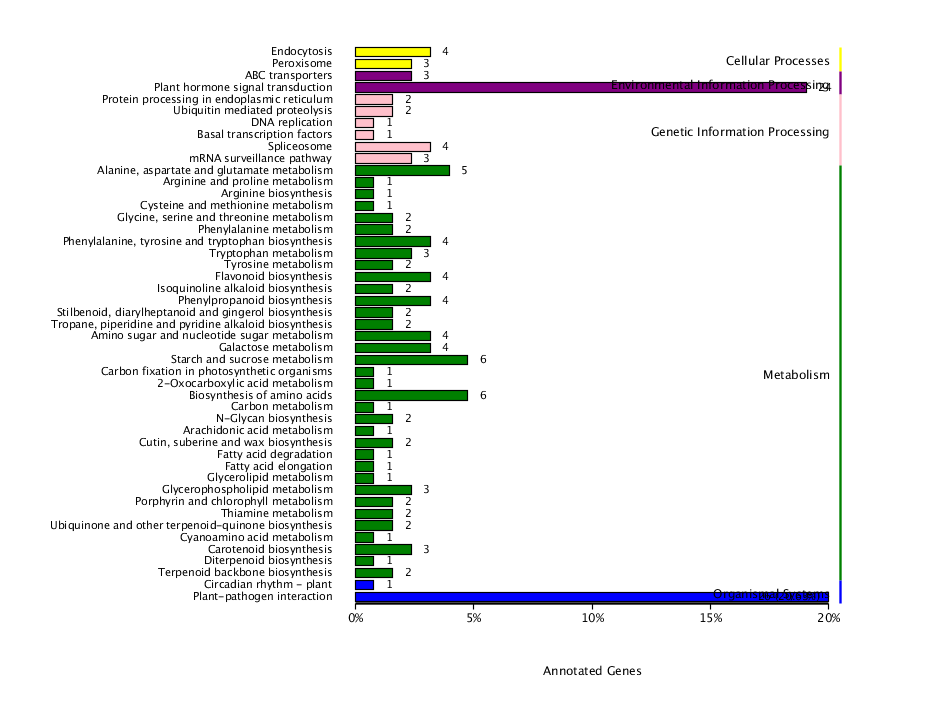


Figure S4. GO (Up) and KEGG (Down) enrichment black module by WGCNA.

a


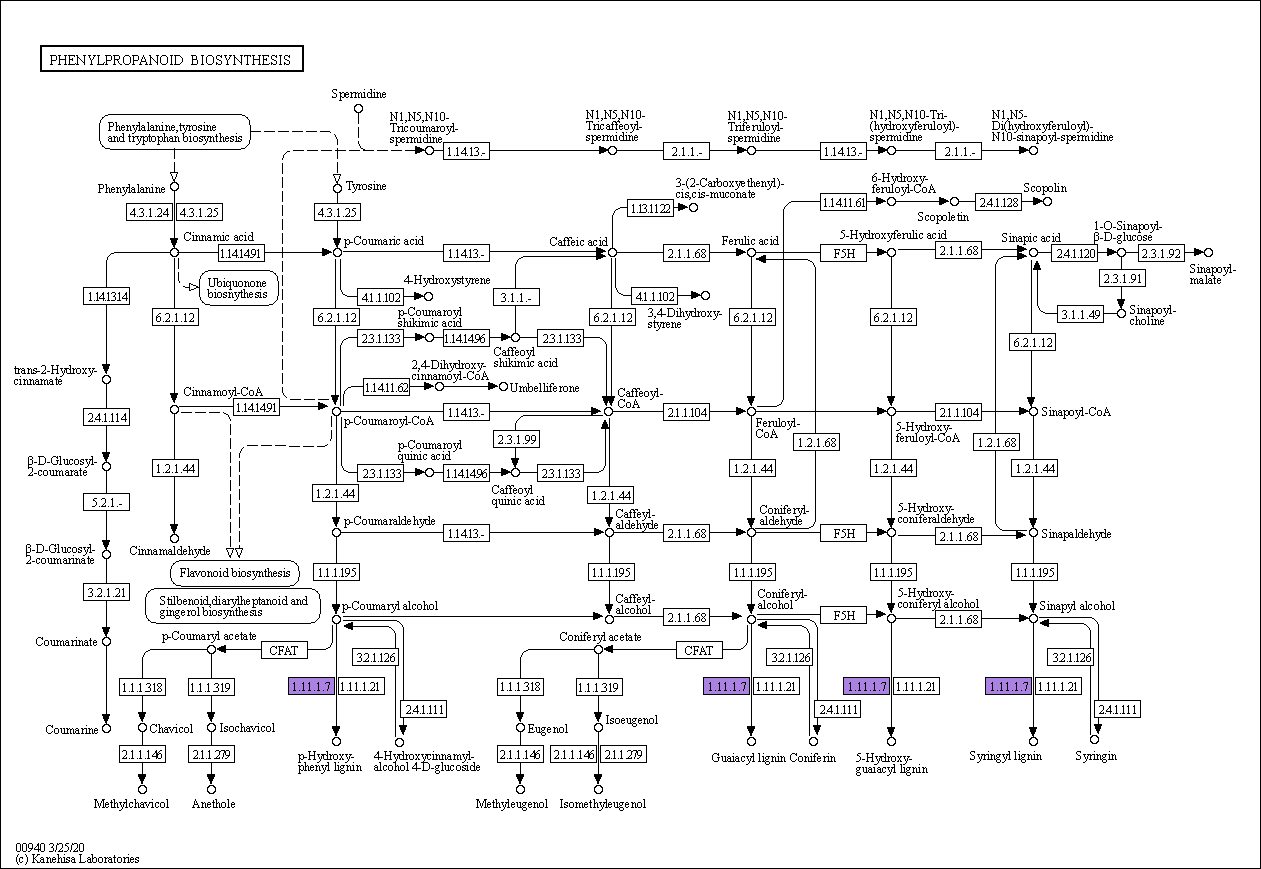


b
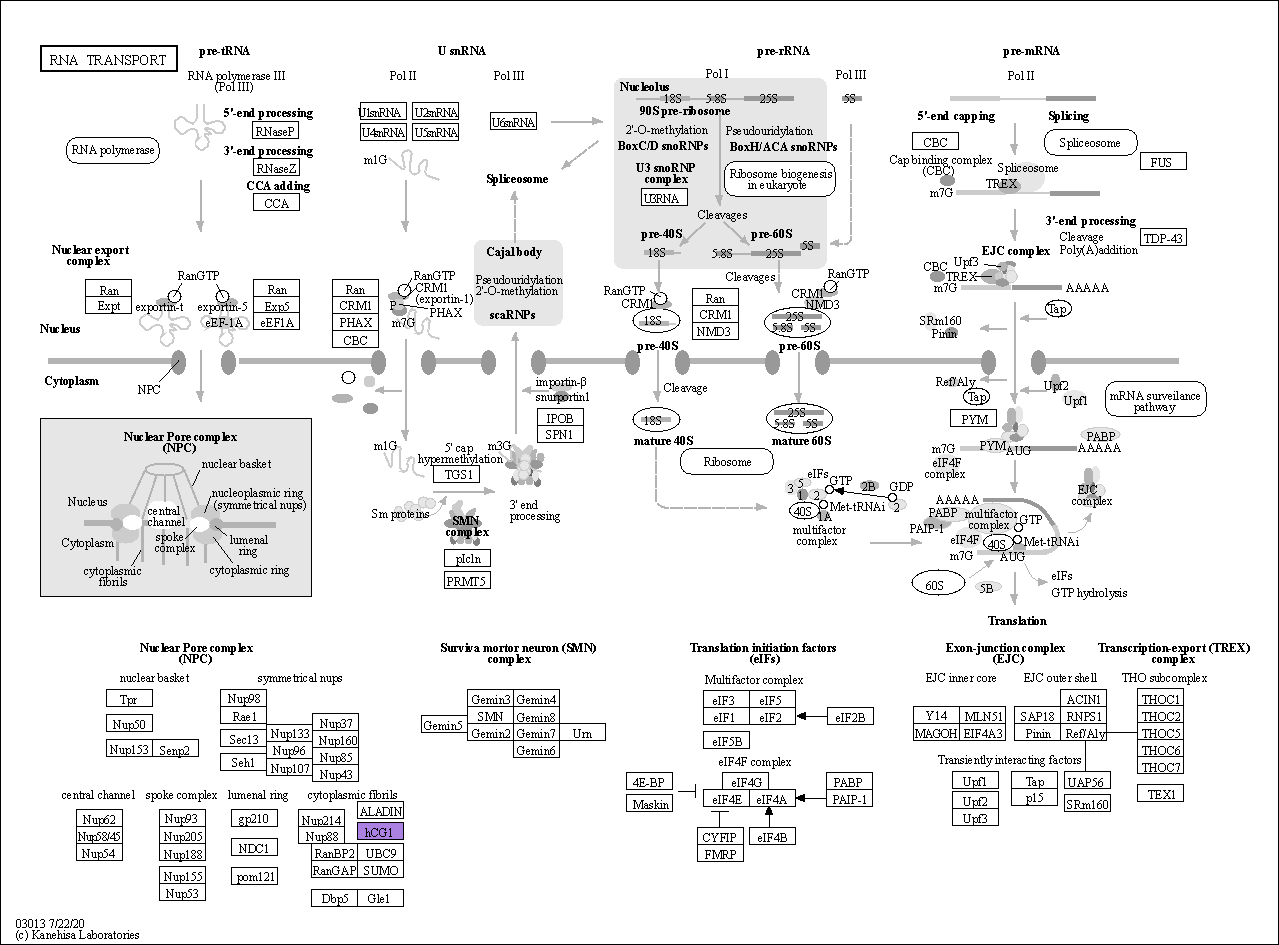


C


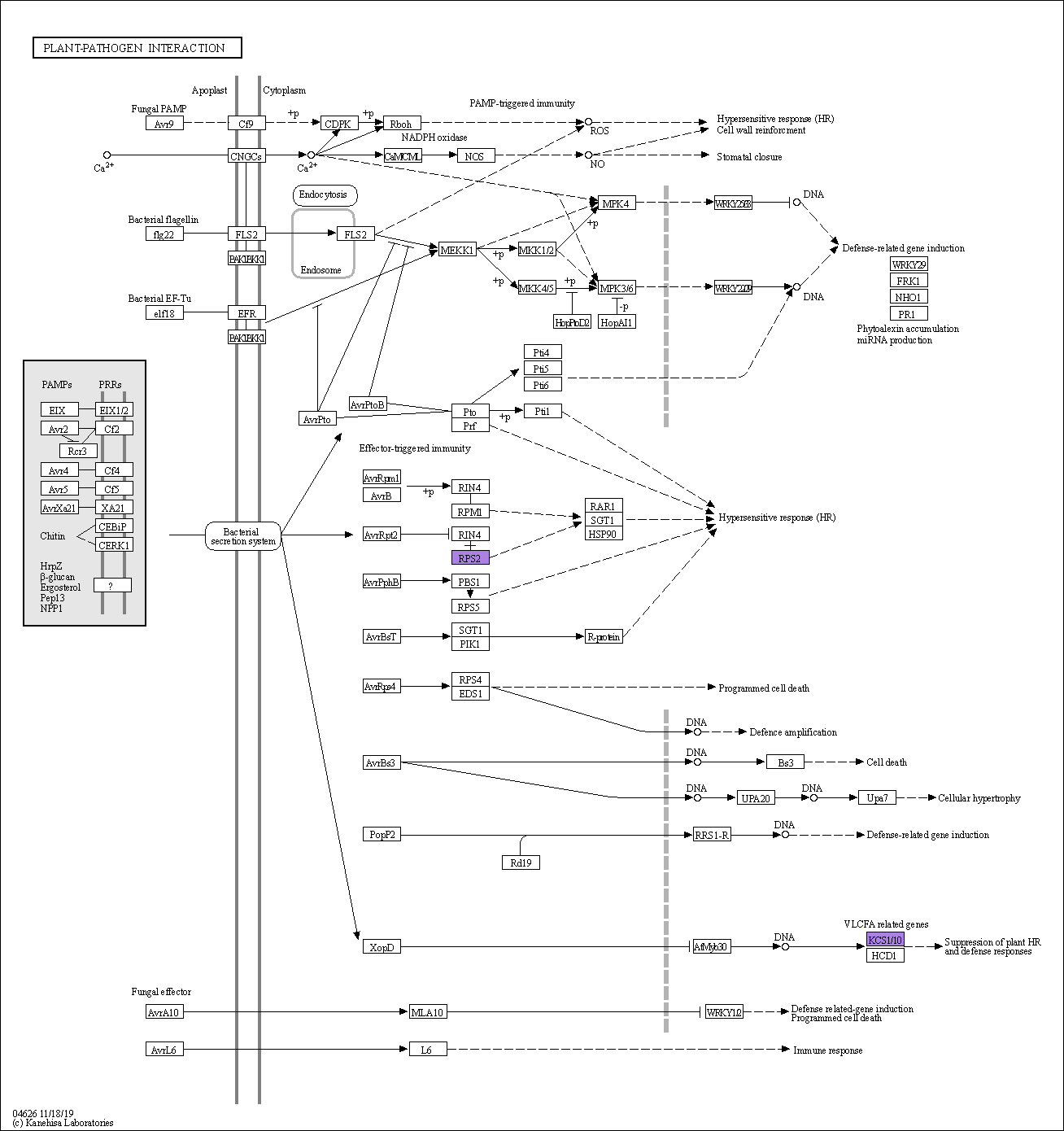


Figure S5. The key pathways Ca for peanut grow. a: ko00940; b: ko03013; c: ko04626.
